# β-Nicotinamide Adenine Dinucleotide (β-NAD) Inhibits ATP-Dependent IL-1β Release from Human Monocytic Cells

**DOI:** 10.3390/ijms19041126

**Published:** 2018-04-10

**Authors:** Sebastian Daniel Hiller, Sarah Heldmann, Katrin Richter, Innokentij Jurastow, Mira Küllmar, Andreas Hecker, Sigrid Wilker, Gabriele Fuchs-Moll, Ivan Manzini, Günther Schmalzing, Wolfgang Kummer, Winfried Padberg, J. Michael McIntosh, Jelena Damm, Anna Zakrzewicz, Veronika Grau

**Affiliations:** 1 Laboratory of Experimental Surgery, Department of General and Thoracic Surgery, Justus-Liebig-University Giessen, German Centre for Lung Research (DZL), Feulgen-Str. 10-12, D-35385 Giessen, Germany; sdhiller@icloud.com (S.D.H.); Sarah.Heldmann@med.uni-giessen.de (S.H.); Katrin.Richter@chiru.med.uni-giessen.de (K.R.); mira.kuellmar@gmx.de (M.K.); Andreas.Hecker@chiru.med.uni-giessen.de (A.H.); sigridwilker@web.de (S.W.); Gabriele.Fuchs-Moll@chiru.med.uni-giessen.de (G.F.-M.); Winfried.Padberg@chiru.med.uni-giessen.de (W.P.); Jelena.Damm@vetmed.uni-giessen.de (J.D.); Anna.Zakrzewicz@chiru.med.uni-giessen.de (A.Z.); 2 Institute of Anatomy and Cell Biology, Justus-Liebig-University Giessen, German Centre for Lung Research (DZL), Aulweg 123, D-35385 Giessen, Germany; innokentij.jurastow@charite.de (I.J.); Wolfgang.Kummer@anatomie.med.uni-giessen.de (W.K.); 3 Department of Animal Physiology and Molecular Biomedicine, Justus-Liebig-University Giessen, Heinrich-Buff-Ring 38, D-35392 Giessen, Germany; Ivan.Manzini@physzool.bio.uni-giessen.de; 4 Institute of Pharmacology and Toxicology, RWTH Aachen University, Wendlingweg 2, D-52072 Aachen, Germany; gschmalzing@ukaachen.de; 5 Department of Biology, University of Utah, 257 South 1400 East, Salt Lake City, UT 84112, USA; mcintosh.mike@gmail.com; 6 George E. Wahlen Veterans Affairs Medical Center, 500 Foothill Drive, Salt Lake City, Salt Lake City, UT 84148, USA; 7 Department of Psychiatry, University of Utah, 501 Chipeta Way, Salt Lake City, UT 84108, USA

**Keywords:** β-NAD, β-nicotinamide adenine dinucleotide, CHRNA7, CHRNA9, CHRNA10, inflammasome, interleukin-1β, iPLA2β, monocyte, P2RY1, P2RY11, PLA2G6, U937 cells

## Abstract

While interleukin-1β (IL-1β) is a potent pro-inflammatory cytokine essential for host defense, high systemic levels cause life-threatening inflammatory syndromes. ATP, a stimulus of IL-1β maturation, is released from damaged cells along with β-nicotinamide adenine dinucleotide (β-NAD). Here, we tested the hypothesis that β-NAD controls ATP-signaling and, hence, IL-1β release. Lipopolysaccharide-primed monocytic U937 cells and primary human mononuclear leukocytes were stimulated with 2′(3′)-*O*-(4-benzoyl-benzoyl)ATP trieethylammonium salt (BzATP), a P2X7 receptor agonist, in the presence or absence of β-NAD. IL-1β was measured in cell culture supernatants. The roles of P2Y receptors, nicotinic acetylcholine receptors (nAChRs), and Ca^2+^-independent phospholipase A2 (iPLA2β, PLA2G6) were investigated using specific inhibitors and gene-silencing. Exogenous β-NAD signaled via P2Y receptors and dose-dependently (IC_50_ = 15 µM) suppressed the BzATP-induced IL-1β release. Signaling involved iPLA2β, release of a soluble mediator, and nAChR subunit α9. Patch-clamp experiments revealed that β-NAD inhibited BzATP-induced ion currents. In conclusion, we describe a novel triple membrane-passing signaling cascade triggered by extracellular β-NAD that suppresses ATP-induced release of IL-1β by monocytic cells. This cascade links activation of P2Y receptors to non-canonical metabotropic functions of nAChRs that inhibit P2X7 receptor function. The biomedical relevance of this mechanism might be the control of trauma-associated systemic inflammation.

## 1. Introduction

Interleukin-1β (IL-1β) is a potent pro-inflammatory cytokine, predominantly but not exclusively produced by innate immune cells, that plays a central role in host defense against infections. The other side of the coin, unfortunately, is the risk of devastating inflammatory diseases such as shock, systemic inflammatory response syndrome, and sepsis, which can be caused by high systemic IL-1β levels [1]. Hence, a tight control of IL-1β production and release is vital. Multiple signals and pathways have been described that trigger the controlled biosynthesis and release of IL-1β. Danger- or pathogen-associated molecular patterns typically activate pattern recognition receptors that induce the biosynthesis of the inactive cytoplasmic precursor pro-IL-1β [2,3,4,5]. Maturation and release of bioactive IL-1β mostly depend on subsequent danger signals that result in assembly of multiprotein complexes, so-called ‘inflammasomes’ [2,3,4,5]. Extracellular ATP released by damaged cells is a well-known trigger of inflammasome activation. Binding of extracellular ATP to the ATP-sensitive P2X7 receptor causes opening of the ion channel, release of K^+^ ions, and assembly of the NLRP3 (NACHT, LRR and PYD domains-containing protein 3) inflammasome followed by activation of caspase-1 that cleaves the inactive pro-IL-1β and enables the release of mature IL-1β [2,4,5,6].

Mechanisms controlling inflammasome activation, in spite of the presence of activating signals, are of outstanding clinical relevance, as progress in this emerging field of research will open up the opportunity for the treatment and prevention of life-threatening inflammatory disorders. Several endogenous control mechanisms seem to be part of negative feedback loops induced by severe inflammation [7,8,9,10] and by fasting, a typical consequence of severe injury and sickness behavior [11,12,13,14]. Previously, we demonstrated that rat monocytes activated during rejection of experimental allografts produce acetylcholine (ACh) [15]. ACh, choline, nicotine and phosphocholine dose-dependently and efficiently inhibit the ATP-induced release of IL-1β by human and rodent monocytic cells via nicotinic ACh receptor (nAChR) subunits α7, α9 and α10 [8,9]. Mao et al. [7] found that over-expression of inducible NO synthase by classically activated macrophages results in nitrosylation and inhibition of NLRP3. Further, the ketone body β-hydroxy-butyrate, a fatty acid metabolite that is increased during fasting or low carbohydrate diet, prevents K^+^ efflux from activated macrophages and by this NLRP3 inflammasome assembly [11].

Fasting also increases cytoplasmic levels of β-nicotinamide adenine dinucleotide (β-NAD) that plays an eminent and well-described role in energy metabolism [16,17]. Intracellular concentrations typically range between 200 and 500 µM, depending on the physiological status and the age of the organism [16,17,18]. Generally speaking, overeating and aging decrease intracellular β-NAD concentrations [19]. Intracellular β-NAD levels regulate NLRP3 inflammasome assembly by at least two distinct mechanisms. β-NAD serves as co-factor for the mitochondrial deacetylase sirtuin-3 (SIRT3) that attenuates NLRP3 inflammasome activation by reducing mitochondrial levels of reactive oxygen species [13]. Likewise, β-NAD-dependent deacetylation of α-tubulin by cytoplasmic sirtuin-2 (SIRT2) inhibits intracellular transport mechanisms involved in NLRP3 inflammasome assembly [12]. Hence, physiological states leading to reduced intracellular β-NAD concentrations promote the release of inflammasome-dependent cytokines.

β-NAD also acts as an extracellular signaling molecule regulating immunity [16,20,21,22,23,24,25,26]. Extracellular β-NAD levels, similar to extracellular ATP levels, are low under steady-state conditions but rise, whenever cells are damaged and cytoplasm is released [27,28]. In addition, ATP and β-NAD are secreted by intact immune cells during acute inflammation [16,17,22]. Several signaling mechanisms of extracellular β-NAD have been described. (1) P2Y1 and P2Y11 receptors interact with G-proteins and mediate metabotropic effects in granulocytes and neurons [21,29]. (2) β-NAD is the substrate of CD38/CD157 that catalyzes the formation of cyclic ADP-ribose or nicotinic adenine dinucleotide phosphate, factors that induce Ca^2+^ signaling [30]. (3) β-NAD is a substrate of the ectoenzyme mono-ADP-ribosyltransferase A2 (ART2) that consumes extracellular β-NAD by catalyzing the covalent transfer of the ADP ribose group from β-NAD onto arginine residues of membrane proteins [23,24,26,31]. Interestingly, ART2-dependent ribosylation of P2X7 receptors expressed by mouse T cells induces long-lasting ATP-independent P2X7 receptor activation followed by T cell apoptosis [23,24,26]. Mouse regulatory T cells and natural killer T cells are particularly sensitive to β-NAD-induced death [26]. However, this mechanism is absent from humans, because the human homolog of mouse ART2 (alias RT6) is non-functional due to a premature stop codon [31].

As ATP and β-NAD are often released together to the extracellular milieu and exert partially overlapping effects as endogenous danger molecules, we tested the hypothesis that extracellular β-NAD affects the ATP-induced maturation and release of IL-1β by human monocytic cells. We describe a novel β-NAD-mediated triple membrane-passing signaling cascade involving P2Y receptors and nAChRs that efficiently suppresses ATP-dependent IL-1β release by human monocytic cells.

## 2. Results

### 2.1. Inhibition of ATP-Mediated Release of IL-1β

**Figure 1:**
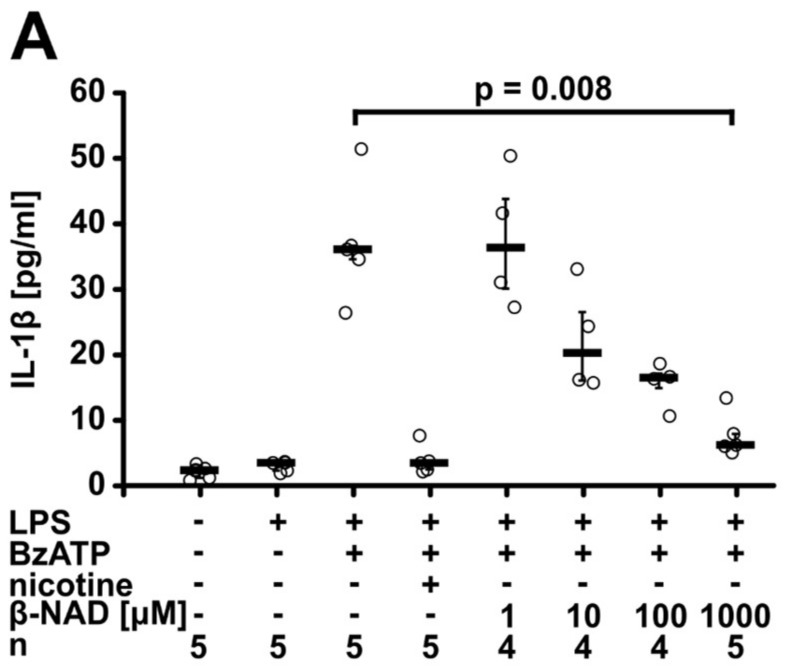

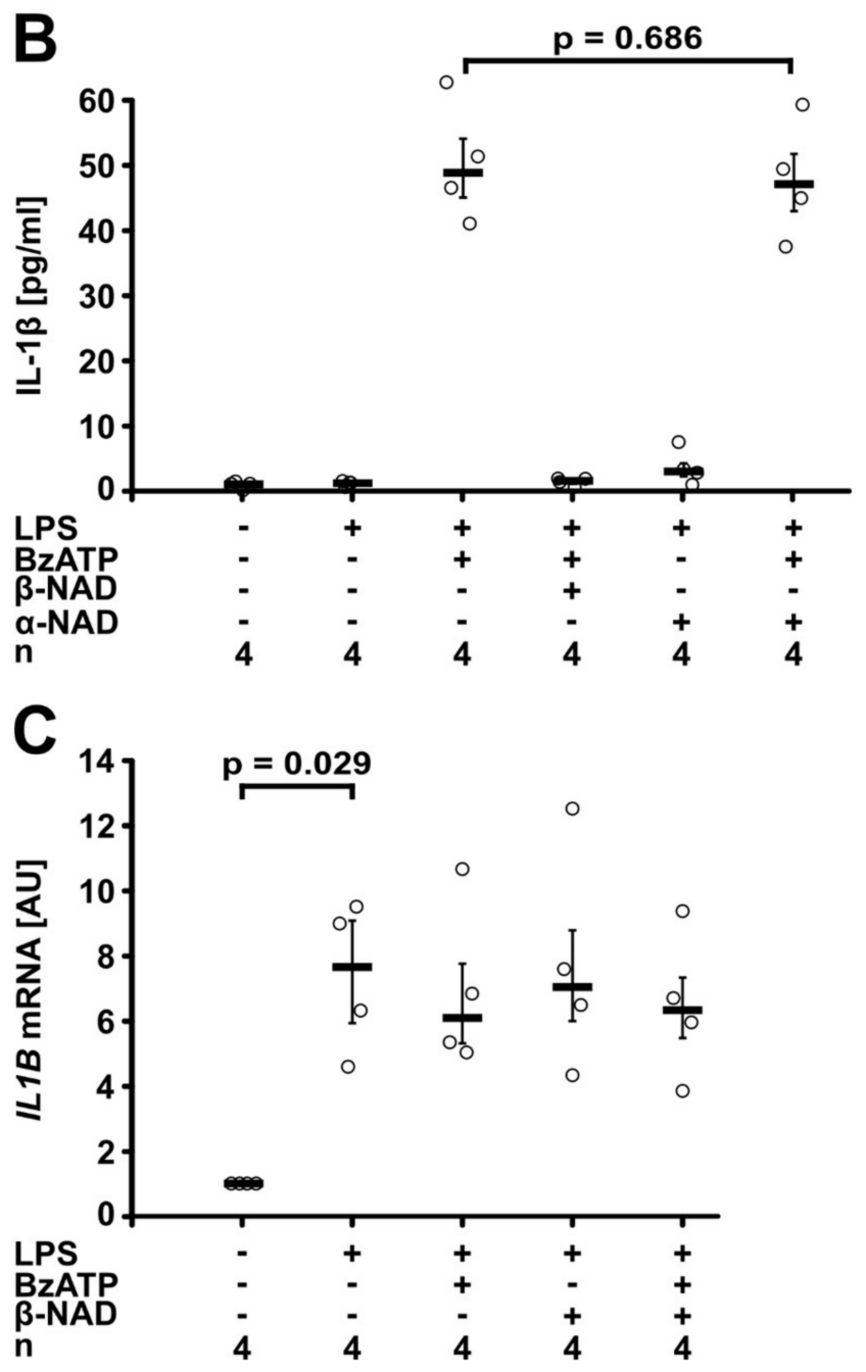
β-nicotinamide adenine dinucleotide (β-NAD) inhibits ATP-induced IL-1β release by U937 cells. (**A**,**B**) Human monocytic U937 cells were primed with lipopolysaccharide (LPS) (1 µg/mL, 5 h) and stimulated with 2′(3′)-*O*-(4-benzoylbenzoyl)adenosine-5′-triphosphate (BzATP; 100 µM, 30 min). The concentration of IL-1β in the cell culture supernatant was measured by ELISA. (**A**) β-NAD dose-dependently inhibited the BzATP-induced release of IL-1β (IC_50_ = 15 µM). Nicotine (10 µM) was included as a positive control. (**B**) In contrast to β-NAD (1 mM), α-NAD (1 mM) was ineffective. (**C**) Real-time RT-PCR was performed to measure the mRNA expression of pro-IL-1β (*IL1B*). The house-keeping gene *HMBS* was included for normalization, data are normalized to the values of untreated U937 cells and are expressed as arbitrary units (AU). Data are presented as individual data points, bars indicate median, whiskers encompass the 25th to 75th percentile, Kruskal-Wallis test followed by the Mann-Whitney rank sum test.

In the first set of experiments, we tested if extracellular β-NAD interferes with the ATP-induced release of IL-1β. Human monocytic U937 cells (10^6^ cells/mL) were primed for 5 h with lipopolysaccharide (LPS; 1 µg/mL) to induce the synthesis of pro-IL-1β followed by addition of 2′(3′)-*O*-(4-benzoyl-benzoyl)ATP trieethylammonium salt (BzATP; 100 µM), a specific agonist of the P2X7 receptor [32], in the presence or absence of different concentrations of β-NAD (1 µM to 1 mM). IL-1β concentrations in the cell culture medium were measured by enzyme-linked immunosorbent assay (ELISA) 30 min later. In line with previous reports [8,9,33], LPS-primed U937 cells spontaneously released almost no IL-1β, but upon stimulation with BzATP, about 40 pg/mL IL-1β were detected in the cell culture supernatant. Nicotine (10 µM), that was included as a positive control [8], fully inhibited the BzATP-induced release of IL-1β. β-NAD dose-dependently (IC_50_ = 15 µM) and efficiently inhibited the BzATP-induced release of IL-1β to the cell culture supernatant (*p* = 0.008, *n* = 5, for 1 mM β-NAD, Figure 1A). While 1 mM β-NAD fully inhibited the release of IL-1β, the same concentration of α-NAD was ineffective (Figure 1B). Cell death as measured by the release of lactate dehydrogenase (LDH) was unimpaired by addition of β-NAD (Figure S1). In all following experiments at best a minor but sometimes significant increase in LDH concentrations was seen (Figures S2–S7). Real-time RT-PCR experiments revealed that the mRNA expression of pro-IL-1β (*IL1B*) was up-regulated by LPS-priming (*p* = 0.029, *n* = 4) and remained unchanged after addition of BzATP, β-NAD or a combination thereof (Figure 1C).

**Figure 2:**
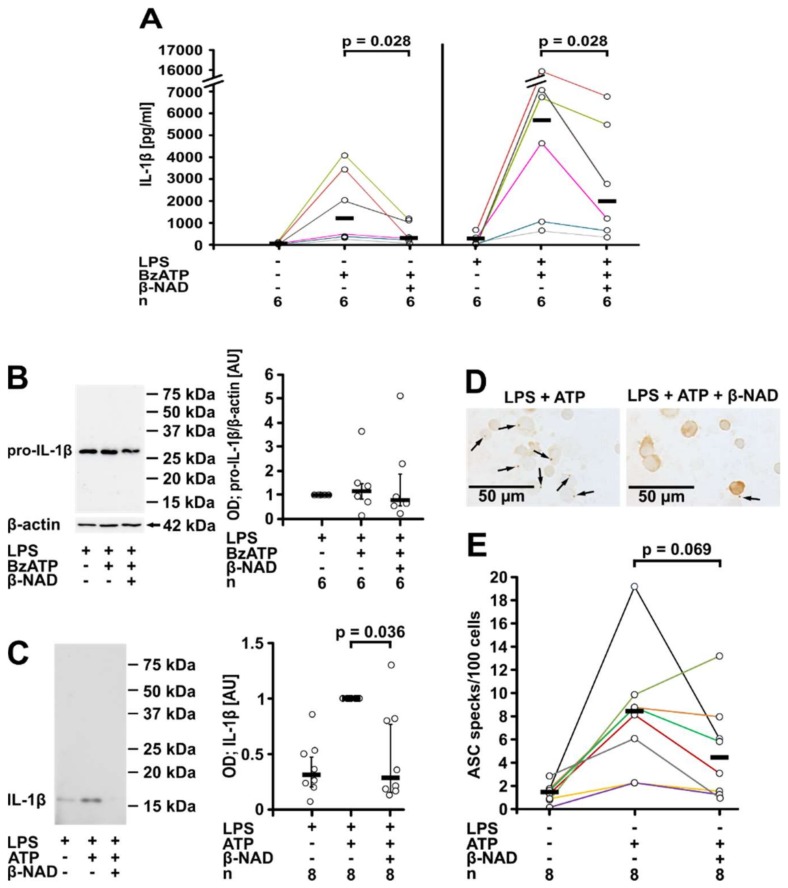
β-NAD inhibits ATP-induced IL-1β release by primary peripheral blood mononuclear leukocytes (PBMCs). (**A**–**C**) PBMCs from healthy donors were left untreated or pulsed with LPS (5 ng/mL) during the process of leukocyte isolation, cultured for 3 h, and stimulated with BzATP (100 µM, 30 min) in the presence or absence of β-NAD (1 mM). (**A**) The concentration of IL-1β was measured in the cell culture supernatant by ELISA. (**B**,**C**) Western blot analysis of cell lysates or concentrated cell culture supernatants using antibodies that recognize pro-IL-1β and mature IL-1β. (**B**) Representative Western blot of cell lysates; pro-IL-1β is detected with an apparent molecular mass of about 34 kDa. A faint signal corresponding to mature IL-1β was obtained in lysates of cells treated with BzATP and β-NAD only in one out of 6 blots. β-actin (40 kDa) was detected on the same blots as a loading control. (**C**) Representative Western blot of cell culture supernatants (one out of 8); only mature IL-1β is detected with an apparent molecular mass of 17 kDa. The optical density (OD) of the immuno-positive bands was measured and the values of the samples from cells stimulated with LPS and BzATP were set to one arbitrary unit (AU). Data are presented as individual data points, bars indicate median, whiskers encompass the 25th to 75th percentile. (**D**,**E**) LPS-pulsed PBMCs were stimulated with ATP (1 mM) and again, β-NAD (1 mM) was added in some experiments. (**D**) ASC immunoreactivity in adherent PBMCs was detected in brown color by immunocytochemistry; arrows are pointing to ASC specks. (**E**) The number of ASC specks per 100 PBMCs was quantified. Data points from individual blood donors are connected by lines in different colors, bars indicate median (**A**,**E**); Wilcoxon signed-rank test.

To test if β-NAD inhibits the BzATP-induced release of IL-1β by primary cells, primary blood mononuclear cells (PBMCs) were either left untreated or shortly pulsed with LPS (5 ng/mL) before cell isolation by gradient centrifugation. The spontaneous secretion of IL-1β by these cells was low as measured by ELISA, whereas a considerable amount of IL-1β was released within 30 min in response to BzATP (100 µM, Figure 2A). β-NAD (1 mM) significantly (*p* = 0.028, *n* = 6, each) attenuated the BzATP-induced release of IL-1β from both untreated and LPS-pulsed PBMCs (Figure 2A). We reported before, that gradient centrifugation and cell handling induces the synthesis of pro-IL-1β in freshly isolated PBMCs, and that almost no IL-18 is secreted by these cells in response to BzATP [8].

Western blot experiments were performed on cell lysates and concentrated cell culture supernatants from LPS-pulsed PBMCs using antibodies that detect both pro-IL-1β and mature IL-1β (Figure 2B,C). Pro-IL-1β with an apparent molecular mass of about 34 kDa was detected in all cell lysates and neither BzATP nor β-NAD significantly changed signal intensity (Figure 2B). A faint band corresponding to mature IL-1β was seen only in one out of 6 experiments. Detection of β-actin (40 kDa) on the same Western blots confirmed equal loading. In contrast, mature IL-1β with an apparent molecular mass of about 17 kDa was detected in the cell culture supernatants and pro-IL-1β remained below the detection limit (Figure 2C). In line with the ELISA data, the results of the quantification of the immunopositive bands showed a low signal in the absence of ATP, a strong increase in response to BzATP and an attenuation of the signal in the presence of β-NAD (*p* = 0.036, *n* = 8, Figure 2C).

Inflammasome formation was assessed by immunocytochemical detection of ASC (apoptosis-associated speck-like protein containing a CARD) specks (Figure 2D,E). In LPS-primed PBMCs staining with antibodies to ASC revealed a diffuse distribution of the immunopositive signal compatible with a nuclear and cytoplasmic distribution of ASC. Only in very few cells, speck-like structures were seen. Stimulation of LPS-primed PBMCs with ATP (1 mM) for 30 min increased the proportion of ASC speck-positive cells, whereas addition of β-NAD (1 mM) tended to reduce speck formation (*p* = 0.069; *n* = 8) (Figure 2D,E). In negative controls, where primary antibodies were omitted, virtually no immunopositivity was seen.

### 2.2. Involvement of Purinergic P2Y Receptors

**Figure 3:**
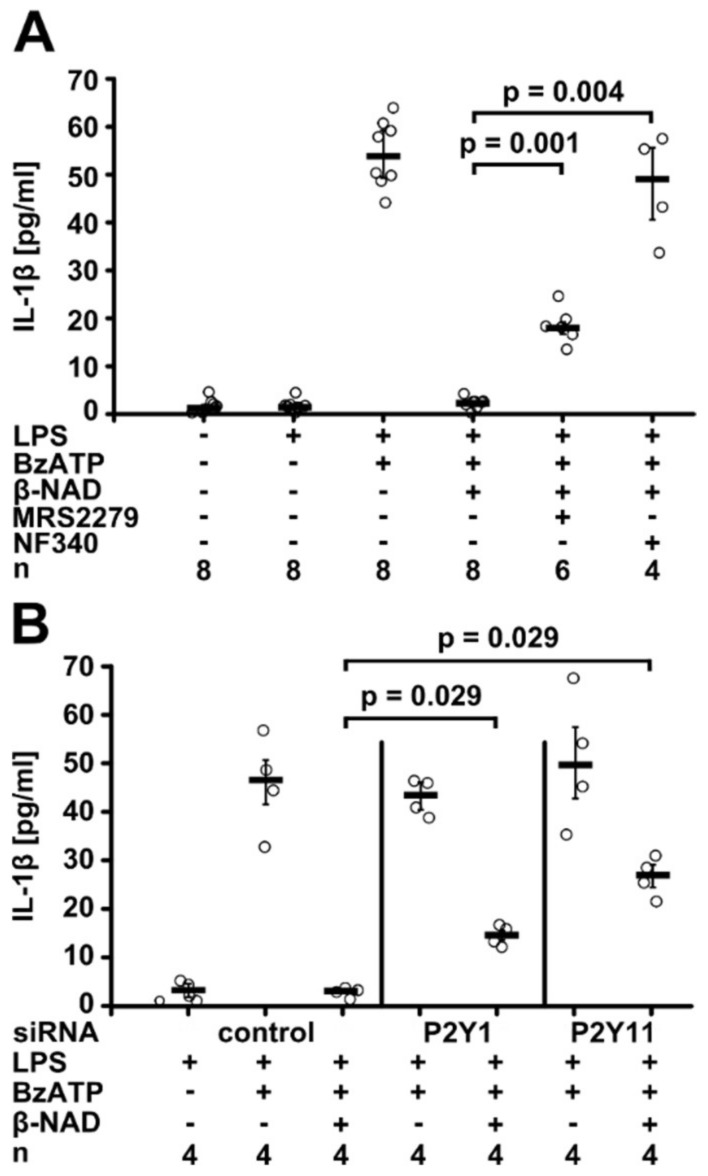
β-NAD signals via P2Y receptors. Human monocytic U937 cells were primed with LPS (1 µg/mL, 5 h) and stimulated with 2′(3′)-*O*-(4-benzoylbenzoyl)adenosine-5′-triphosphate (BzATP; 100 µM, 30 min). β-NAD was applied together with BzATP at a concentration of 1 mM. The concentration of IL-1β in the cell culture supernatant was measured by ELISA. (**A**) Experiments were performed in the presence or absence of the P2Y1 receptor antagonist MRS2279 (500 nM) or of the P2Y11 receptor antagonist NF340 (5 µM); (**B**) U937 cells were transfected with control siRNA or with siRNA targeting P2Y1 or P2Y11 (*P2RY1*, *P2RY11*). Data are presented as individual data points, bars indicate median, whiskers encompass the 25th to 75th percentile, Kruskal-Wallis test followed by the Mann-Whitney rank sum test.

Next, we tested if purinergic P2Y receptors are involved in β-NAD signaling. In the presence of MRS2279 (500 nM), an antagonist of the purinergic P2Y1 receptor [34], the inhibitory effect of β-NAD (1 mM) was attenuated to about one third of the total IL-1β release (*p* = 0.001, *n* = 8 vs. *n* = 6, Figure 3A). Strikingly, the P2Y11 receptor antagonist NF340 (5 µM) [35], fully restored the BzATP-dependent release of IL-1β by LPS-primed U937 cells in spite of the presence of β-NAD (*p* = 0.004, *n* = 8 vs. *n* = 4, Figure 3A). In the absence of BzATP, neither P2Y receptor antagonist provoked the release of IL-1β by LPS-primed U937 cells, and these antagonists did not change the amount of IL-1β released in response to BzATP (Figure S8). Western blotting demonstrated expression of both receptors in U937 cells (Figure S9A,C).

In support of the inhibitor experiments, gene silencing of P2Y1 and P2Y11 receptor (*P2RY1, P2RY11*) expression in U937 cells by small interfering RNA (siRNA) transfection impaired the inhibitory effect of β-NAD (1 mM) on IL-1β release (*p* = 0.029, *n* = 4, Figure 3B), whereas treatment with control siRNA was ineffective. Efficient silencing of P2Y1 and P2Y11 receptor protein expression was validated by Western blotting (Figure S9A–D).

### 2.3. Involvement of iPLA2β

**Figure 4:**
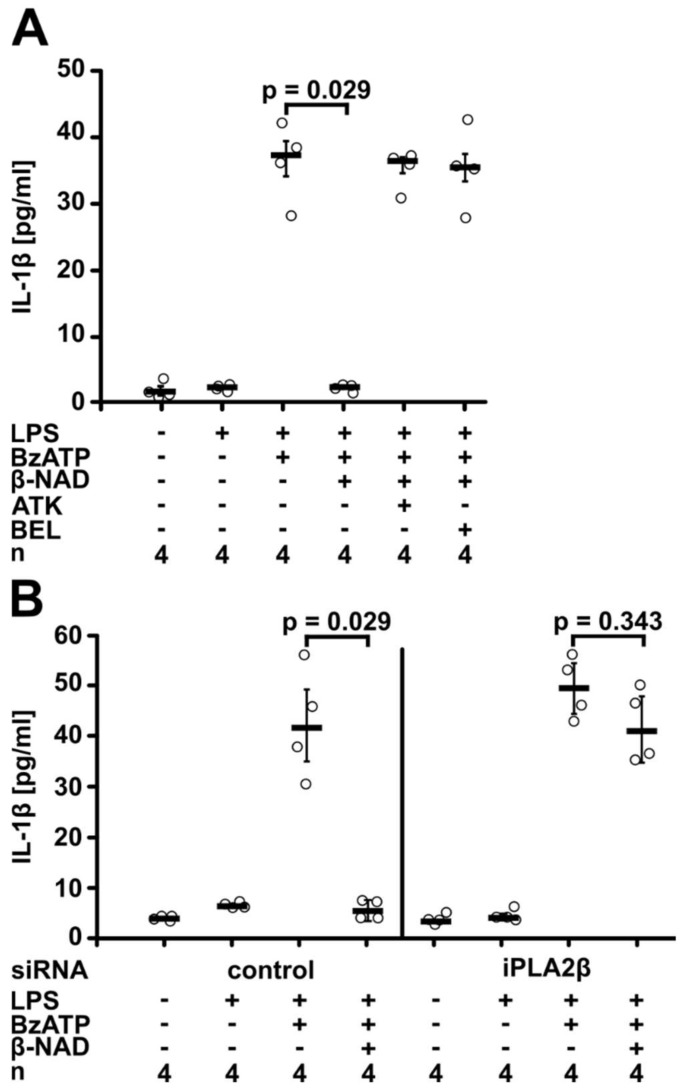
β-NAD signaling involves iPLA2β. Human monocytic U937 cells were primed with LPS (1 µg/mL, 5 h) and activated with 2′(3′)-*O*-(4-benzoylbenzoyl)adenosine-5′-triphosphate (BzATP; 100 µM, 30 min). β-NAD was used at a concentration of 1 mM. The concentration of IL-1β in the cell culture supernatant was measured by ELISA. (**A**) Experiments were performed in the presence or absence of the general inhibitor of PLA2 arachidonyl trifluoromethyl ketone (AKT; 50 µM) or of the more specific iPLA2 inhibitor bromoenol lactone (BEL; 50 µM). (**B**) U937 cells were transfected with control siRNA or with siRNA targeting iPLA2β (*PLA2G6*). Data are presented as individual data points, bars indicate median, whiskers encompass the 25th to 75th percentile, Kruskal-Wallis test followed by the Mann-Whitney rank sum test.

As P2Y receptor signaling can lead to the activation of phospholipase A2 (PLA2) [36,37,38], we wondered if PLA2 is involved in β-NAD signaling in our experimental setting. We used arachidonyl trifluoromethyl ketone (ATK; 50 µM), an inhibitor of Ca^2+^-dependent PLA2 and Ca^2+^-independent PLA2 (iPLA2) [39,40], and bromoenol lactone (BEL; 50 µM), a more specific inhibitor of iPLA2β [40]. Both inhibitors fully reversed the effect of β-NAD (1 mM) (*p* = 0.029, *n* = 4, Figure 4A). When LPS-primed U937 cells were treated with ATK or BEL in the absence of BzATP, virtually no IL-1β was released into the cell culture medium, and these inhibitors did not change the amount of IL-1β released in response to BzATP (Figure S8). Accordingly, gene silencing of iPLA2β (*PLA2G6*) abolished the β-NAD effect (*n* = 4, Figure 4B). We recently confirmed by Western blotting in the same experimental setting, that iPLA2β is expressed by U937 cells, and that transfection of specific siRNA significantly reduces iPLA2β protein levels [41].

### 2.4. Release of a Soluble Factor

**Figure 5:**
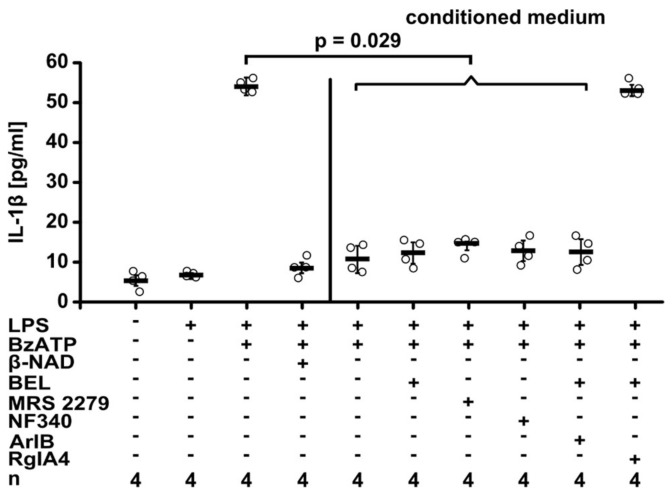
β-NAD triggers the release of a bioactive soluble factor. Human monocytic U937 cells were primed with LPS (1 µg/mL, 5 h) and treated with β-NAD (1 mM) for 30 min to produce conditioned medium. Thereafter, the conditioned medium was harvested and applied to another set of LPS-primed U937 cells together with 2′(3′)-*O*-(4-benzoylbenzoyl)adenosine-5′-triphosphate (BzATP; 100 µM) and incubated for 30 min. The P2Y1 antagonist MRS2279 (500 nM), the P2Y11 antagonist NF340 (5 µM), the iPLA2 inhibitor bromoenol lactone (BEL; 50 µM), the α7 nAChR antagonist α-conotoxin ArIB [V11L, V16D] (500 nM), or the α9α10 nAChR antagonist α-conotoxin RgIA4 (200 nM) were applied together with the conditioned medium. β-NAD (1 mM) was included in this experiment as a positive control in the absence of conditioned medium. The release of IL-1β to the cell culture supernatant was measured by ELISA. Data are presented as individual data points, bars indicate median, whiskers encompass the 25th to 75th percentile, Kruskal-Wallis test followed by the Mann-Whitney rank sum test.

Activated iPLA2β typically cleaves membrane phosphatidylcholines and eventually leads to the secretion of diverse lipid mediators [36,37,38]. Hence, we postulated that a bioactive factor is released into the cell culture medium in response to stimulation with β-NAD. LPS-primed U937 cells were incubated for 30 min with β-NAD (1 mM) to produce a conditioned medium containing the postulated mediator. Thereafter, conditioned medium was applied together with BzATP to another set of LPS-primed U937 cells. To prevent a direct effect of β-NAD, conditioned medium was tested in the presence of the P2Y1 receptor antagonist NF340 (5 µM), the P2Y11 antagonist MRS2279 (500 nM) or of the iPLA2β inhibitor BEL (50 µM). In line with the hypothesis that β-NAD induces the release of a bioactive factor, conditioned medium of LPS-primed cells treated with β-NAD fully inhibited the BzATP-induced release of IL-1β, despite the presence of NF340, MRS2279 or BEL (*p* = 0.029, *n* = 4, Figure 5).

### 2.5. Involvement of nAChRs

**Figure 6:**
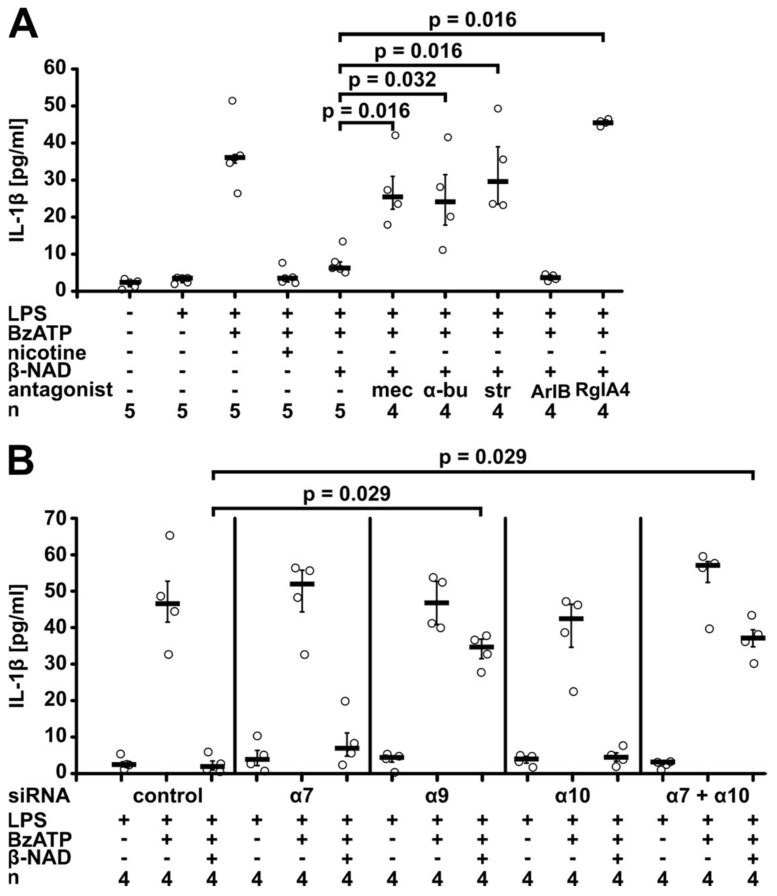
β-NAD signaling involves nicotinic acetylcholine receptors. Human monocytic U937 cells were primed with LPS (1 µg/mL, 5 h) and activated with 2′(3′)-O-(4-benzoylbenzoyl)adenosine-5′-triphosphate (BzATP; 100 µM, 30 min). β-NAD was applied together with BzATP at a concentration of 1 mM. The concentration of IL-1β in the cell culture supernatant was measured by ELISA. (**A**) Nicotinic antagonists mecamylamine (mec; 100 µM), α-bungarotoxin (α-bu; 1 µM), strychnine (str; 10 µM), and α-conotoxin RgIA4 (200 nM) reversed the inhibitory effect of β-NAD, whereas α-conotoxin ArIB [V11L, V16D] (500 nM) was ineffective; (**B**) U937 cells were transfected with control siRNA or with siRNA targeting nicotinic acetylcholine receptor subunits α7, α9, or α10 (*CHRNA7, CHRNA9, CHRNA10*). Data are presented as individual data points, bars indicate median, whiskers encompass the 25th to 75th percentile, Kruskal-Wallis test followed by the Mann-Whitney rank sum test.

We demonstrated before, that ATP-dependent release of IL-1β by human and murine monocytic cells can be inhibited by stimulation of nAChRs containing subunits α7, α9 and α10 [8,9,10]. In view of these findings, we hypothesized that the signaling cascade activated by β-NAD also involves nAChRs. If so, the inhibitory effect of β-NAD (1 mM) should be sensitive to nAChR antagonists. Indeed, in the presence of mecamylamine (100 µM), a general inhibitor of nAChRs, β-NAD was fully inactive in suppressing the BzATP-induced release of IL-1β by LPS-primed U937 cells (*p* = 0.016, *n* = 5 vs. *n* = 4, Figure 6A). The effect of β-NAD was also reversed by α-bungarotoxin (1 µM; *p* = 0.032, *n* = 5 vs. *n* = 4, Figure 6A), an antagonist of nAChRs containing subunits α7 or α9, and by strychnine (10 µM; *p* = 0.016, *n* = 5 vs. *n* = 4, Figure 6A) that more selectively antagonizes subunit α9-containing nAChR [42,43,44]. To differentiate between nAChR containing subunits α7 or α9, we used specific conotoxins ArIB [V11L, V16D] (500 nM) or RgIA4 (200 nM) [9,45,46]. The inhibitory effect of β-NAD was fully antagonized by the α9-specific conotoxin RgIA4 (*p* = 0.016, *n* = 4 vs. *n* = 5), whereas ArIB was ineffective (Figure 6A). Likewise, the activity of conditioned medium from β-NAD-treated LPS-primed U937 was sensitive to RgIA4 (*p* = 0.029, *n* = 4) and not to ArIB (Figure 5). We showed previously, that all nAChR antagonists used in this study do not induce the release of IL-1β by LPS-primed U937 cells, when given in the absence of BzATP [8,9,33].

To further corroborate the involvement of nAChRs in β-NAD signaling, U937 cells were transfected with siRNA targeting mRNA for nAChR subunits α7, α9 and α10 (*CHRNA7, CHRNA9, CHRNA10*) either alone or in combination. The specificity and efficiency of the siRNAs targeting subunits α9 and α10 in U937 cells was recently shown by our group in the same experimental setting; only the mRNA expression of subunit α7 was too low for quantification [8,10]. Unfortunately, it is not possible to specifically detect these nAChR receptor subunits on the protein level due to a lack of antibodies specific for human nAChR [47,48]. Silencing of nAChR subunit α9 restored the BzATP-induced release of IL-1β to about 75% in spite of the presence of β-NAD (1 mM) (*p* = 0.029, *n* = 4, Figure 6B), whereas individual silencing of α7 or α10 did not impair the effect of β-NAD. In contrast, when the expression of nAChR subunits α7 and α10 was silenced concomitantly, the effect of β-NAD was blunted (*p* = 0.029, *n* = 4, Figure 6B) similar to the single knock-down of subunit α9. Double knock-down of nAChR subunits α7 or α10 together with subunit α9 (*p* = 0.029, *n* = 4) or silencing of all three subunits (*n* = 3) resulted in similar effects to silencing of subunit α9 alone (Figure S10).

### 2.6. Inhibition of P2X7 Receptor Function

**Figure 7:**
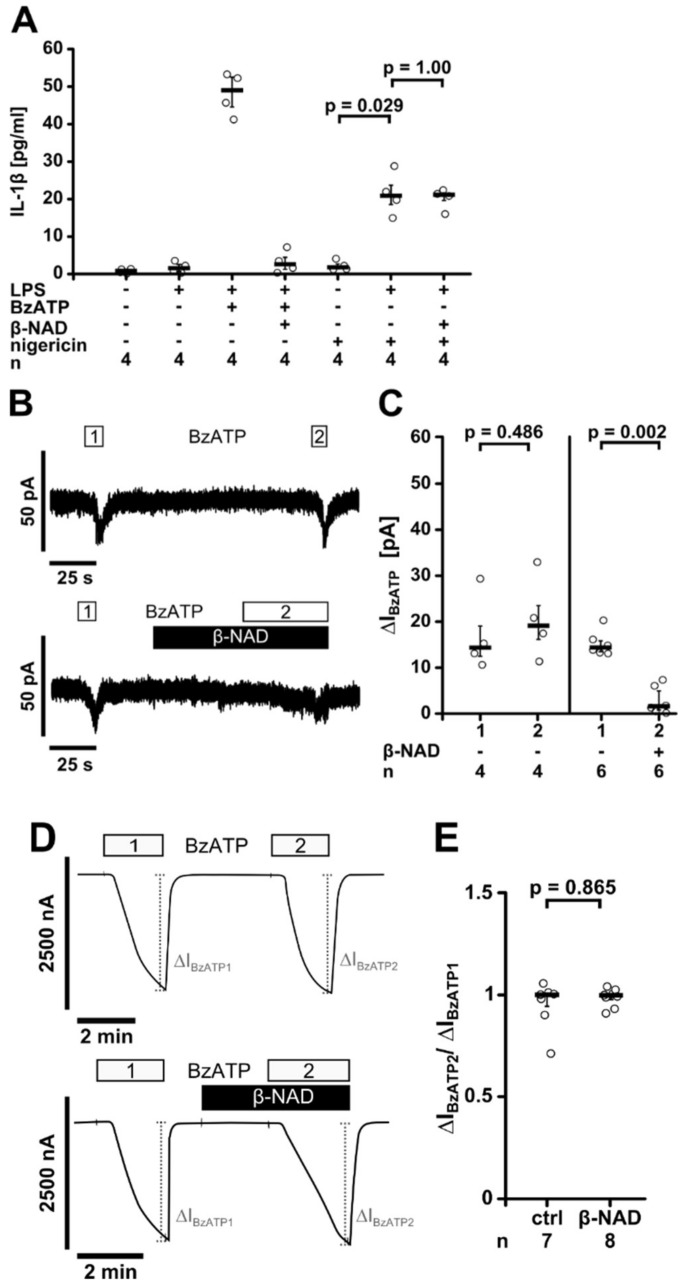
β-NAD inhibits the ion channel function of ATP receptor P2X7. (**A**) Human monocytic U937 cells were primed with LPS (1 µg/mL, 5 h) and activated with 2′(3′)-*O*-(4-benzoylbenzoyl)adenosine-5′-triphosphate (BzATP; 100 µM, 30 min) or nigericin (50 µM) in combination with apyrase (0.5 U/mL). β-NAD was used at a concentration of 1 mM. The concentration of IL-1β in the cell culture supernatant was measured by ELISA; (**B**,**C**) BzATP-induced ion currents were detected by whole-cell patch-clamp measurements in LPS-primed U937 cells; (**B**) Repetitive current changes provoked by two (1, 2) consecutive BzATP (100 µM) applications. Application of β-NAD (1 mM) alone did not provoke ion currents but fully inhibited the response to BzATP; (**C**) Graphical presentation of the two consecutive BzATP-induced ion current changes (1, 2, ΔI_BzATP_) (**D**,**E**) Two-electrode voltage-clamp (TEVC) measurements were performed on *Xenopus laevis* oocytes that heterologously expressed human P2X7 receptors; (**D**) Representative current curves. In control experiments (ctrl; upper panel) BzATP (10 µM; 2 min) induced repetitive stimulations of the transmembrane ion current (∆I_BzATP1_ and ∆I_BzATP2_). Application of β-NAD (1 mM) had no impact on ΔI_M_ and did not impair the consecutive response to BzATP (lower panel); (**E**) Normalized ∆I_BzATP_ values (∆I_BzATP2_/∆I_BzATP1_) from experiments as shown in (**D**). Data are presented as individual data points, bar represents median, whiskers encompass the 25th to 75th percentile (**A**,**C**,**E**). Experimental groups were compared by or Kruskal-Wallis followed by Mann-Whitney rank sum test (**A**) or Wilcoxon signed-rank test (**C**,**E**).

Next, we investigated if extracellular β-NAD inhibits ATP-independent NLRP3 inflammasome assembly induced by the pore-forming toxin nigericin. As expected, nigericin (50 µM) induced the release of IL-1β from LPS-primed U937 cells at a slightly lesser extent than BzATP [8,33]. Nigericin was applied together with the ATP-degrading enzyme apyrase (0.5 U/mL) to prevent possible confounding effects of endogenous ATP. In this experimental setting, β-NAD (1 mM) did not impair the nigericin-induced release of IL-1β (*n* = 4, Figure 7A).

We reported recently, that classical and unconventional agonists of monocytic nAChRs inhibited BzATP-induced ion currents via metabotropic signaling [8,9,10]. To test the hypothesis that β-NAD impairs the ionotropic P2X7 receptor function, whole-cell patch-clamp experiments were performed on LPS-primed U937 cells. As expected, BzATP induced repeatable ion currents due to P2X7 receptor activation (Figure 7B,C) [8,9,10]. When β-NAD (1 mM) was applied to LPS-primed U937 cells, no changes in ion currents occurred. In line with our hypothesis, the ion current responses to BzATP were virtually abolished in the presence of β-NAD (*p* = 0.002, *n* = 6, Figure 7C).

To investigate if β-NAD directly interferes with the ionotropic P2X7 receptor function, we used *Xenopus laevis* oocytes as a heterologous expression system of human P2X7 receptors and performed two-electrode voltage-clamp (TEVC) experiments. As expected, application of BzATP (10 µM) evoked responses of the transmembrane ion current (∆I_M_) that were reversible and repeatable (Figure 7D,E). BzATP-induced currents were unimpaired in the presence of β-NAD (1 mM) (*p* = 0.865, Figure 7D,E), suggesting that β-NAD does not inhibit P2X7 receptor function.

## 3. Discussion

We demonstrate in this study that β-NAD efficiently and dose-dependently inhibits BzATP-induced release of IL-1β by human monocytic cells. Signaling involves purinergic P2Y receptors, activation of iPLA2β, and release of a factor with a low molecular mass that functions as a nAChR agonist. Stimulation of nAChRs containing subunit α9 inhibits the ion channel function of the ATP-sensitive P2X7 receptor, and hence, impairs ATP-induced IL-1β maturation and release.

Our evidence that extracellular β-NAD inhibits BzATP-dependent inflammasome activation in human monocytic cells is manifold. Upon application of β-NAD to LPS-primed monocytic U937 cells, the release of IL-1β to the cell culture supernatant is dose-dependently and fully inhibited. β-NAD is as effective as nicotine that functions as a strong inhibitor of ATP-dependent IL-1β release from human monocytic cells, as described previously [8]. The inhibitory effect on IL-1β release is also present, albeit not as pronounced, in adherent primary human PBMCs obtained from male volunteers. All blood donors reported that they were healthy non-smokers, and no other inclusion or exclusion criteria were applied. We can only speculate about the reason why β-NAD does not fully inhibit the BzATP-induced release of IL-1β by PBMCs. Apart from monocytes, PBMCs also contain lymphocytes, natural killer cells and dendritic cells that might not respond to β-NAD like U937 cells. Furthermore, blood monocytes from healthy donors can be divided into at least three different subpopulations [49] and it remains to be investigated if they differ in their responsiveness towards β-NAD.

We set out to investigate the signaling cascade that links extracellular β-NAD to the inhibition of IL-1β release. Several signaling pathways of β-NAD that have already been described can be considered. High intracellular β-NAD concentrations exert protective functions and can indeed control the activation of the NLRP3 inflammasome [12,13,50]. However, typical cytoplasmic concentrations of β-NAD are at least one order of magnitude above the here described IC_50_ of exogenous β-NAD [16,17,18]. Rapid uptake of β-NAD as a function of concentration has not been previously described. Likewise, the uptake of high concentrations of exogenous β-NAD is slow, as intracellular concentrations peak 6 h after application [50]. In contrast, we demonstrated that β-NAD immediately and fully inhibits the BzATP-induced IL-1β release, a process that starts within minutes. Hence, it is unlikely, that in our experimental setting an increase in intracellular β-NAD concentrations is responsible for the observed inhibitory effect. Another option would be CD38/CD157-dependent conversion of β-NAD to the Ca^2+^-mobilizing factors cyclic ADP-ribose or nicotinic acid adenine dinucleotide phosphate [30]. However, human monocytes mount Ca^2+^ responses to extracellular β-NAD independent of CD38 [51], making an involvement of at least CD38 in the β-NAD signaling unlikely.

We investigated if signaling of β-NAD is mediated via purinergic P2Y1 and/or P2Y11 receptors, as reported previously by several authors for diverse cell types [20,21,25,52,53,54]. P2Y1 and P2Y11 receptors are membrane receptors of ATP and β-NAD that, depending on their interacting G proteins, induce diverse signaling cascades. P2Y1 receptors typically activate phospholipase C and the release of Ca^2+^ from intracellular stores, whereas P2Y11 receptors increase both, intracellular Ca^2+^ concentrations and cAMP levels [51,52,53,54,55]. Ligand specificity, down-stream signaling and receptor internalization upon agonist binding are modified by heterooligomer formation of P2Y1 and P2Y11 receptors [36,54,56]. We are only beginning to discover the potential diversity of P2Y receptor responses.

In human monocytes, β-NAD induces Ca^2+^ signals via engagement of P2Y1 and P2Y11 receptors [54]. Using inhibitors of P2Y receptors, we provide evidence that the inhibitory effect of β-NAD on the BzATP-induced release of IL-1β is mediated via the P2Y11 receptor and at least in part also via the P2Y1 receptor. These data were corroborated in experiments, in which U937 cells were transfected with siRNA targeting the expression of P2Y1 or P2Y11 receptors. The effect of β-NAD on the release of IL-1β was, however, not fully reversed, which might be due to the incomplete inhibition of receptor expression that was evidenced by Western blotting. While β-NAD (1 mM) fully inhibited the BzATP-induced release of IL-1β, the same concentration of α-NAD was ineffective. This result is in contrast to studies on human granulocytes, where α- and β-NAD induced virtually identical Ca^2+^ signals via the P2Y11 receptor [21]. At present, we cannot explain this obvious discrepancy between granulocytes and monocytic cells.

The conundrum remains that extracellular ATP and BzATP are classical agonists of P2Y1 and P2Y11 receptors [55] and, hence, should both induce and inhibit the release of IL-1β, but this is obviously not the case. Similar to our results, the effects of β-NAD on smooth muscle cells depend on the P2Y1 receptor, but are not elicited by ATP [29]. We can only speculate about the reasons for this discrepancy. Heterooligomer formation of P2Y11 with other P2Y receptors can change the agonist preference as shown for P2Y11/P2Y1 receptor heteromers [56], although up to now, there is no report on such a preference for β-NAD over BzATP.

How does activation of purinergic receptors by β-NAD link to the inhibition of BzATP-mediated IL-1β release? P2Y receptors can activate PLA2 via G proteins either directly or via activation of phospholipase C, production of inositol triphosphate, Ca^2+^ signaling, depletion of intracellular Ca^2+^ stores, and store-operated Ca^2+^ re-entry [36,37,38]. Here we demonstrate that iPLA2β is involved in the β-NAD-induced signaling cascade by two independent approaches. β-NAD-dependent inhibition of IL-1β release is sensitive to inhibitors of iPLA2β and is almost abrogated upon silencing of iPLA2β. We conclude from our results that iPLA2β is an essential component of the signaling cascade induced by β-NAD.

The next obvious question is how activation of iPLA2β translates into an inhibition of IL-1β release. Activated iPLA2β preferentially cleaves membrane phosphatidylcholines to form free fatty acids, predominantly arachidonic acid, which is the precursor of numerous bioactive oxylipins. In addition, lysophosphatidylcholine is formed that can be further metabolized to glycerophosphocholine, phosphocholine, and choline [57]. We demonstrated before, that compounds containing a phosphocholine-group and choline itself can inhibit ATP-dependent IL-1β release by monocytes [8,9,10,33]. Therefore, we hypothesized that activation of iPLA2β results in the secretion of soluble bioactive mediators. Indeed, small molecules are released by U937 cells in response to β-NAD, mediating the inhibition of IL-1β release independent of receptors P2Y1 or P2Y11 and independent of iPLA2β. We are currently elucidating the chemical identity of this compound or mixture of compounds.

Due to the substrate preference of iPLA2β and the fact that phosphocholine-containing compounds are unconventional nAChR agonists in monocytes, we speculated that β-NAD activates the formerly described cholinergic pathway that controls the ionotropic function of P2X7 receptors and hence, inflammasome assembly and IL-1β release [8,9,10,33]. Both our pharmacological studies and gene-silencing experiments suggest that nAChR subunit α9 is essential for this signaling pathway, whereas subunits α7 and α10 are not. The observation that the effect of β-NAD was blunted after double knock-down of nAChR subunits α7 and α10 deserves further investigation. The nAChR subunit requirements for the signaling of β-NAD differ from those described for nicotine, acetylcholine, phosphocholine, glycerophosphocholine or palmitoyl-lysophosphatidylcholine [8,9,10], but are similar to those for the surfactant lipid component dipalmitoyl-phosphatidylcholine (DPPC) [33].

Recently, we described a similar mechanism active in human monocytic cells that is triggered by the chemokine CCL3 [41]. CCL3 signals via the G protein-coupled receptor CCR1, activates iPLA2β, leads to the secretion of a nAChR agonist of low molecular mass, and, finally, to the inhibition of BzATP-induced IL-1β release. In contrast to the here described mechanism induced by β-NAD, all three nAChR subunits α7, α9 and α10 are needed for the inhibition of IL-1β release by CCL3 [41], suggesting that different mediators are secreted. The different nAChR subunit requirements might justify the speculation that the mediator released in response to CCL3 is a rather small metabolite of phosphatidylcholines such as phosphocholine, whereas the by β-NAD-induced mediator is a more complex or more lipophilic compound [8,9,10,33].

Finally, we confirmed the hypothesis that β-NAD indirectly inhibits BzATP-induced ion currents in patch-clamp experiments, suggesting that β-NAD inhibits the ionotropic response of the ATP-sensitive receptor P2X7. In line with these results, ATP-independent stimulation of IL-1β release with the pore-forming bacterial toxin nigericin was not influenced by β-NAD. Of note, β-NAD did not induce ion currents when applied to LPS-primed U937 cells. As TEVC measurements on *Xenopus oocytes* revealed that β-NAD does not impair the ionotropic function of P2X7 receptors, we conclude that the effect of β-NAD is indirect. A similar inhibition of the ionotropic P2X7 receptor function was shown before for the classical nAChR agonists choline and nicotine, as well as for the unconventional nAChR agonists, phosphocholine, glycerophosphocholine, lysophosphatidylcholine and DPPC [8,9,10,33]. We conclude that β-NAD leads to nAChR activation that efficiently inhibits ionotropic functions of P2X7 receptors. Interestingly, depending on the species, extracellular β-NAD can either activate P2X7 receptors and induce cell death as shown for mouse lymphocytes [23,24,26] or inhibit P2X7 receptor activation as demonstrated here for human monocytic cells. In line with the inhibition of the ionotropic P2X7 receptor function, β-NAD did not induce the release of larger amounts of the cytoplasmic enzyme LDH.

This novel mechanism controlling ATP-induced monocytic IL-1β secretion, depends on β-NAD concentrations (IC_50_ = 15 µM), that can be expected in the extracellular milieu during severe inflammation and after mechanical tissue injury [22], suggesting that β-NAD can also control IL-1β release in vivo. As ATP is frequently released along with β-NAD, it is of interest to compare their relative cytoplasmic concentrations as well as their efficiencies in activating the ATP-sensitive P2X7 receptor (EC_50_ of ATP: 2–4 mM) and in inhibiting IL-1β release (IC_50_ of β-NAD: 15 µM), to estimate their net effect. As cytoplasmic β-NAD concentrations typically range between 200 and 500 µM [16,17,18] and ATP levels between 1 and 10 mM [58], it can be predicted that the ATP-induced release of IL-1β is inhibited, when primed monocytes are surrounded by freshly spilled cytoplasm of injured cells. However, the net effect in vivo will also depend on the local metabolism of ATP- and β-NAD that is difficult to predict.

Our study has several limitations. Although we discovered an anti-inflammatory pathway induced by β-NAD that might be of high biological and clinical relevance, we can only outline the signaling cascade from the binding of β-NAD to purinergic receptors to the inhibition of IL-1β release: (1) It is unclear, why the anti-inflammatory effect is triggered by β-NAD but seemingly not by BzATP. (2) We did not elucidate the mechanisms down-stream of P2Y receptor activation if iPLA2β is activated directly or via intracellular Ca^2+^ signals. (3) The chemical identity of the potential nAChR agonist that is released upon stimulation with β-NAD remains to be identified. (4) The inhibitory interaction of nAChRs and ATP receptor P2X7 is still enigmatic. (5) The crucial question if β-NAD also prevents IL-1β release in vivo remains to be answered. (6) Finally, we do not know which cell types in addition to blood monocytes react to β-NAD in the described way.

**Figure 8:**
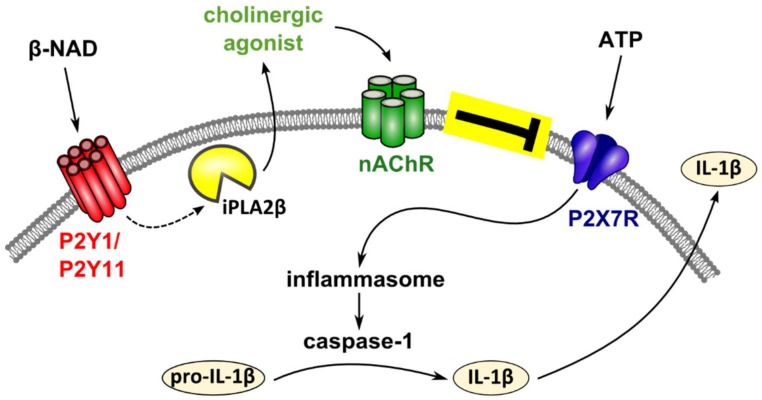
Schematic presentation of the proposed mechanism. Extracellular β-NAD and ATP are spilled concomitantly from damaged cells. ATP binds to the ATP-sensitive P2X7 receptor of monocytic cells, induces inflammasome activation, activation of caspase-1, cleavage of pro-IL-1β and release of bioactive IL-1β. Our data suggest that β-NAD signals via P2Y receptors that activate iPLA2β and result in the production and secretion of yet unidentified bioactive mediators. These mediators seem to function as nicotinic agonists that activate non-canonical metabotropic functions at nicotinic acetylcholine receptors. Nicotinic receptor stimulation, in turn, efficiently inhibits the ion-channel function of the P2X7 receptor. It is unclear, if nicotinic receptors subunits actually form conventional pentamers as shown in the schematic drawing.

In conclusion, we discovered a novel anti-inflammatory mechanism of extracellular β-NAD that leads to an efficient inhibition of the release of IL-1β by human monocytic cells (Figure 8). We provide evidence that β-NAD signals via P2Y receptors that activate iPLA2β, resulting in the production and secretion of yet unidentified bioactive mediators, probably unconventional nAChR agonists. These factors activate non-canonical metabotropic functions at nAChRs containing subunit α9 that, in turn, efficiently inhibit the ion-channel function of the P2X7 receptor. This triple membrane-passing signaling cascade eventually inhibits ATP-dependent release of monocytic IL-1β. With all due caution, we suggest that this anti-inflammatory pathway might control the life-threatening excessive release of IL-1β in response to tissue injury. Certainly much more evidence, primarily in vivo data, is needed to verify or refute this assumption.

## 4. Materials and Methods

### 4.1. U937 Cells

U937 cells, a human histiocytic lymphoma cell line, were obtained from the German Collection of Microorganisms and Cell Cultures (Braunschweig, Germany) and cultured in RPMI 1640 (Gibco by Life Technologies, Darmstadt, Germany), 10% fetal calf serum (FCS; Biochrome, Berlin, Germany), 2 mM l-glutamine (Gibco), at 37 °C, 5% CO_2_. Cells were seeded in 24-well plates (10^6^ cells/mL) and primed with 1 μg/mL LPS from *Escherichia coli* (L2654, Sigma-Aldrich, Taufkirchen, Germany) for 5 h. Thereafter, U937 cells were stimulated for 30 min with the P2X7 receptor agonist BzATP (Jena Bioscience, Jena, Germany; 100 µM) and cell culture supernatants were stored at −20 °C. In some experiments, nigericin (50 µM; Sigma-Aldrich) was used as a stimulus of NLRP3 inflammasome activation in the presence of apyrase (0.5 U/mL; Sigma-Aldrich). IL-1β concentrations were measured by Quantikine^®^ Immunoassays (R&D Systems, Minneapolis, MN, USA) and cell death was estimated by measuring lactate dehydrogenase (LDH) using the CytoTox 96^®^ Non-Radioactive Cytotoxicity Assay (Promega, Madison, WI, USA). β-NAD was applied at different concentrations (Sigma-Aldrich; 1–1000 µM) together with BzATP or nigericin/apyrase, in the presence or absence of nicotinic antagonists mecamylamine hydrochloride (100 µM; Sigma-Aldrich), α-bungarotoxin (1 µM; Tocris Bioscience, Bristol, UK), strychnine hydrochloride (10 µM; Sigma-Aldrich), or of the conotoxins ArIB [V11L, V16D] (500 nM) or RgIA4 (200 nM) [9,45,46,59]. P2Y1 and P2Y11 receptors were antagonized by NF340 (Tocris via R&D Systems; 5 µM) and MRS 2279 (Tocris via R&D Systems, 500 nM), respectively. PLA2 was inhibited with ATK (Enzo Life Sciences, Lausen, Switzerland; 50 µM) or BEL (Enzo Life Sciences, Lausen, Switzerland; 50 µM). In some experiments, nicotine (Sigma-Aldrich; 10 µM) was included as a positive control for the inhibition of BzATP-mediated IL-1β release [8].

### 4.2. RNA Isolation and Real-Time RT-PCR

Total RNA was isolated from U937 cells at the end of the experiment and real-time RT-PCR was performed to estimate quantitative changes in pro-IL-1β (*IL1B*) mRNA expression in comparison to the house-keeping gene *HMBS* as described before [8]. In negative control experiments where the cDNA was replaced by water, no amplicons were produced. Data were analyzed using the 2^Δ*C*t^ method. Δ*C*t is the difference between the *C*t value of the house-keeping gene and the gene of interest. Data obtained for untreated control cells were set to 1 arbitrary unit, all other values were calculated accordingly.

### 4.3. PBMCs

All studies on primary human cells were approved by the local ethics committee at the University of Giessen (approval No. 81/13). Blood was drawn from according to their own statements healthy, male, non-smoking adult volunteers into sterile syringes containing 17.5 IU heparin (Ratiopharm, Ulm, Germany) per mL blood and PBMCs were separated on Leucosep gradients (Greiner Bio-One, Frickenhausen, Germany). In some experiments, LPS (5 ng/mL) was added to blood samples before gradient centrifugation. PBMCs were cultured for 3 h in 24-well plates at a density of 5 × 10^5^ cells/0.5 mL in RPMI 1640, 10% FCS, 2 mM l-glutamine. Non-adherent cells were removed, and cell culture medium was replaced by medium devoid of FCS. PBMCs were stimulated with BzATP in the presence or absence of β-NAD as described for U937 cells.

### 4.4. Immunocytochemistry

Immunohistochemistry for the detection of ASC-specks was essentially performed as described before [33]. In contrast to our previous study, ATP (Sigma-Aldrich) was used instead of BzATP in these experiments. In brief LPS-pulsed PBMC were stimulated with ATP (1 mM) in the presence of absence of β-NAD for 30 min followed by fixation in Cytofix/Cytoperm^TM^ (BD Biosciences, Heidelberg, Germany). After blocking endogenous peroxidase activity and unspecific protein binding sites, primary rabbit anti-ASC antibodies (Santa Cruz sc-22514-R, Santa Cruz, CA, USA) were applied and detected by horseradish peroxidase-labeled goat anti-rabbit Ig antibodies (DAKO, Hamburg, Germany) and 3,3′-diaminobenzidine (Sigma-Aldrich). Absence of unspecific background staining caused by secondary antibodies was tested by omission of primary antibodies. Slides were lightly counter-stained with hemalum and evaluated with an Olympus (Hamburg, Germany) BX51 microscope and the analySIS software (Olympus Camedia, version 4.10). At least 1200 cells were counted per experiment.

### 4.5. Gene Silencing

The Amaxa^®^ Cell Line Nucleofector^®^ Kit C from Lonza (Cologne, Germany) was used to transfect U937 cells with *CHRNA7* (nAChRα7)*, CHRNA9* (nAChRα9)*, CHRNA10* (nAChRα10)*, P2RY1 (*P2Y1), *P2RY11* (P2Y2) or *PLA2G6* (iPLA2β) human siRNA ON-TARGETplus SMARTpool (GE Dharmacon, Lafayette, CO, USA), at a concentration of 30 pmol siRNA per 1 × 10^6^ cells. As a negative control, the ON-TARGETplus Non-targeting Control Pool (GE, Dharmacon) was included in each experiment. Cells were cultured for two days before studying the control of BzATP-induced IL-1β release.

### 4.6. Western Blotting

Protein extracts of U937 cells were separated on 12% SDS-polyacrylamide gels under reducing conditions along with dual color precision plus protein standards (Bio-Rad, Hercules, CA, USA) and transferred onto Immobilon polyvinylidene difluoride membranes (Millipore, Billerica, MA, USA). Membranes were blocked with 5% BSA diluted in PBS and incubated with polyclonal rabbit antibodies to P2Y1, P2Y11 (LS-C163318; 1:1000 and LS-C200442; 1:500; LifeSpan Biosciences via Biozol, Eching, Germany) or mouse monoclonal antibodies to β-actin (1:50000; A2228, Sigma-Aldrich). Primary antibodies were detected with horse radish peroxidase-labeled goat anti-rabbit Ig and rabbit anti-mouse Ig secondary antibodies (1:5000; Dako, Glostrup, Denmark), Lumi-Light substrate (Roche, Mannheim, Germany) and High Performance Chemiluminescence Films (GE Healthcare Bio-Sciences, Uppsala, Sweden). Documentation and densitometry were performed using a digital gel documentation system (Biozym, Hessisch Oldendorf, Germany). Western blot analysis of IL-1β in concentrated cell culture supernatants was performed as described before [8].

### 4.7. Production and Use of Conditioned Medium

For the production of conditioned medium, U937 cells were primed with LPS in the absence of FCS, followed by treatment with β-NAD (1 mM) for 30 min. Cells were spun down (500 g) and the supernatant was harvested. 80% of the medium of another set of LPS-primed U937 cells was replaced by conditioned medium and BzATP (100 µM) was added in the absence or presence of BEL, NF340, MRS 2279, RgIA4, or ArIB.

### 4.8. Patch-Clamp Experiments on U937 Cells

Whole-cell patch-clamp recordings on LPS-primed U937 cells were essentially performed as described before [8]. U937 cells were grown in poly-L-lysine coated cell culture dishes containing bath solution (in mM: 5.4 KCl, 120 NaCl, 2 CaCl_2_, 1 MgCl_2_, 25 glucose and 10 HEPES (4-(2-hydroxyethyl)-piperazine-1-ethanesulfonic acid); pH 7.4). First, BzATP (100 µM) was applied via a pressure-driven microperfusion system, followed by β-NAD (1 mM) and by a second BzATP stimulus. To control the reversibility and repeatability of the BzATP-induced changes in ion currents, BzATP was applied twice in the absence of β-NAD at least once per experimental day.

### 4.9. Heterologous Expression of Human P2X7 Receptor in Xenopus Laevis Oocytes and TEVC Measurements

Defolliculated *Xenopus laevis* oocytes (Ecocyte Bioscience, Castrop-Rauxel, Germany) from two different individuals were stored at in oocyte Ringer’s solution (ORi) containing (in mM) 90 NaCl (Fluka, Deisenhofen, Germany), 1 KCl (Fluka), 2 CaCl_2_ (Fluka), 5 HEPES (Sigma-Aldrich), 2.5 pyruvate (Fluka), 20 mg/mL penicillin (Sigma-Aldrich) and 25 mg/mL streptomycin (Sigma-Aldrich) (pH 7.4). Capped cRNA of the human P2X7 receptor was synthesized as described before [60,61] dissolved in Tris/HCl and injected into oocytes (0.35 ng per oocyte) using a microinjector (Nanoject, Drummond Scientific, Broomall, PA, USA). Oocytes were incubated at 17 °C for 1–3 days, before placing them in a perfusion chamber combined with a gravity-driven perfusion system. TEVC measurements were performed as described before [62]. In brief, oocytes were perfused with a Ca^2+^-free Ringer’s solution containing (in mM) 100 NaCl, 2.5 KCl, 5 HEPES and 0.1 flufenamic acid (pH 7.4). The membrane was voltage clamped to −40 mV using a TEVC amplifier (Warner Instruments, Hamden, CT, USA). The I_M_ were low-pass filtered at 1000 Hertz (Frequency Devices 902, Haverhill, MA, USA) and recorded with a strip chart recorder (Kipp & Zonen, Delft, The Netherlands). BzATP (10 µM; 2 min)-induced I_M_ responses were examined in absence and presence of β-NAD (1 mM).

### 4.10. Statistical Analyses

Non-parametric tests were used for statistical analyses, because the relatively low n-numbers typical for cell culture experiments do not allow for precise tests for normal distribution. Results are presented as individual data points, median, and percentiles 25 and 75. Data were analyzed by Wilcoxon signed-rank test or by non-parametric Kruskal-Wallis test followed by the Mann-Whitney rank sum test using the SPSS software (IBM Spss statistics, version 23, Munich, Germany). Paired data were analyzed by Wilcoxon sign rank test (SPSS software). *p*-Values below 0.05 were considered as statistically significant. The IC_50_ value of β-NAD in human U937 cells was determined using GraphPad Prism^®^ (Version 6, GaphPad Software, La Jolla, CA, USA) by fitting log-transformed concentration values and the original effect data.

## Supplementary Materials

Supplementary Materials can be found at http://www.mdpi.com/1422-0067/19/4/1126/s1.

## Abbreviations

IL-1β	interleukin-1β
β-NAD	β-nicotinamide adenine dinucleotide
BzATP	2′(3′)-*O*-(4-benzoyl-benzoyl)ATP trieethylammonium salt
nAChR	nicotinic acetylcholine receptor
PLA2	phospholipase A2
iPLA2β	Ca^2+^-independent phospholipase A2
NLRP3	NACHT, LRR and PYD domains-containing protein 3
ACh	acetylcholine
SIRT3	sirtuin-3
SIRT2	sirtuin-2
ART2	mono-ADP-ribosyltransferase A2
LPS	lipopolysaccharide
LDH	lactate dehydrogenase
ATK	arachidonyl trifluoromethyl ketone
BEL	bromoenol lactone
