# Supplementary material for: β-Nicotinamide Adenine Dinucleotide (β-NAD) Inhibits ATP-Dependent IL-1β Release from Human Monocytic Cells
Source: Int J Mol Sci. 2018 Apr 10;19(4):1126. doi: 10.3390/ijms19041126 (PMC5979475; doi:10.3390/ijms19041126)
Supplement: Supplementary file 1 [file ijms-19-01126-s001.pdf]

Supplementary Materials: β-nicotinamide adenine dinucleotide (β-NAD) inhibits ATP-dependent inflammasome activation in human monocytic cells

Sebastian Daniel Hiller, Sarah Heldmann, Katrin Richter, Innokentij Jurastow, Mira Küllmar, Andreas Hecker, Sigrid Wilker,Gabriele Fuchs-Moll, Ivan Manzini, Günther Schmalzing, Wolfgang Kummer, Winfried Padberg, J. Michael McIntosh, Jelena Damm, Anna Zakrzewicz, & Veronika Grau

Supplemental Figures S1-S10

Figure S1

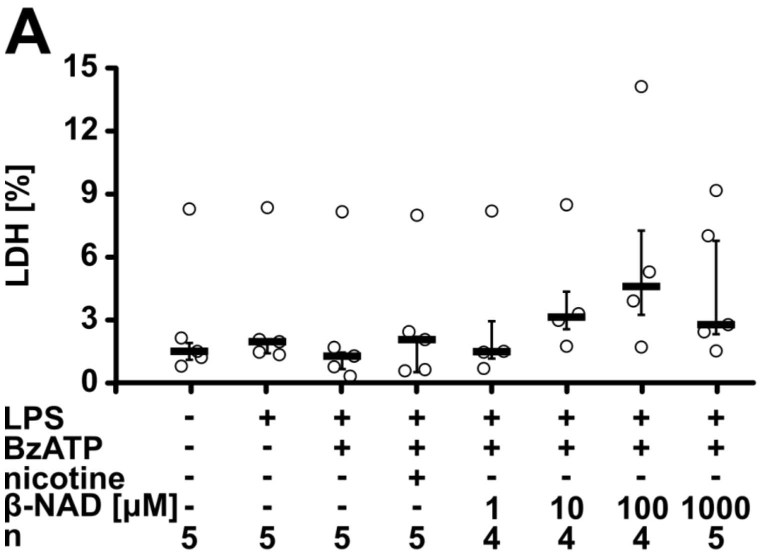

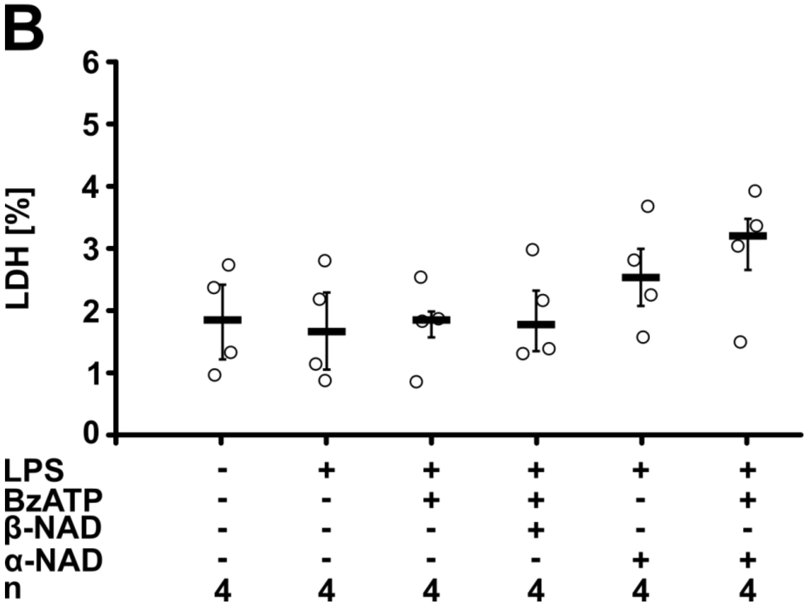

Figure S1. Estimation of cell death in experiments depicted in Figure 1. A, B) Human monocytic U937 cells were primed with LPS (1 µg/ml, 5 h) and stimulated with 2'(3')-O-(4-benzoylbenzoyl)adenosine-5'-triphosphate (BzATP; 100 µM, 30 min). The effects of nicotine (10 µM), β-NAD (1 mM) or α-NAD (1 mM) were investigated. The concentration of the cytoplasmic enzyme lactate dehydrogenase (LDH) was measured in the cell culture supernatants at the end of the experiments. LDH concentrations are indicated in % of the total cellular content of LDH in LPS-primed control cells. No significant differences were induced by β-NAD ($p > 0.05$). Data are presented as individual data points, bars indicate median, whiskers encompass the 25th to 75th percentile, Kruskal-Wallis test followed by the Mann-Whitney rank sum test.

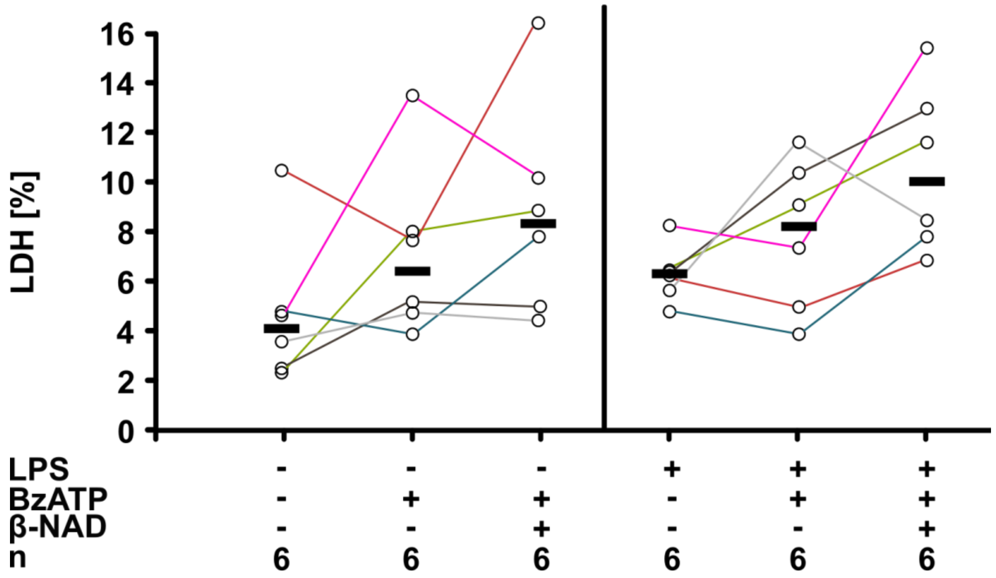

Figure S2. Estimation of cell death in experiments depicted in Figure 2A. The concentration of the cytoplasmic enzyme lactate dehydrogenase (LDH) was measured in the cell culture supernatants at the end of the experiments. LDH concentrations are indicated in % of the total cellular content of LDH in untreated control cells. Peripheral blood mononuclear cells from healthy donors were left untreated or pulsed with LPS (5 ng/ml) during the process of leukocyte isolation, cultured for 3 h, and stimulated with BzATP (100 µM, 30 min) in the presence or absence of β-NAD (1 mM). No significant differences were induced by β-NAD ($p > 0.05$). Data points from individual blood donors are connected by lines in different colors, bars indicate median; Wilcoxon signed-rank test.

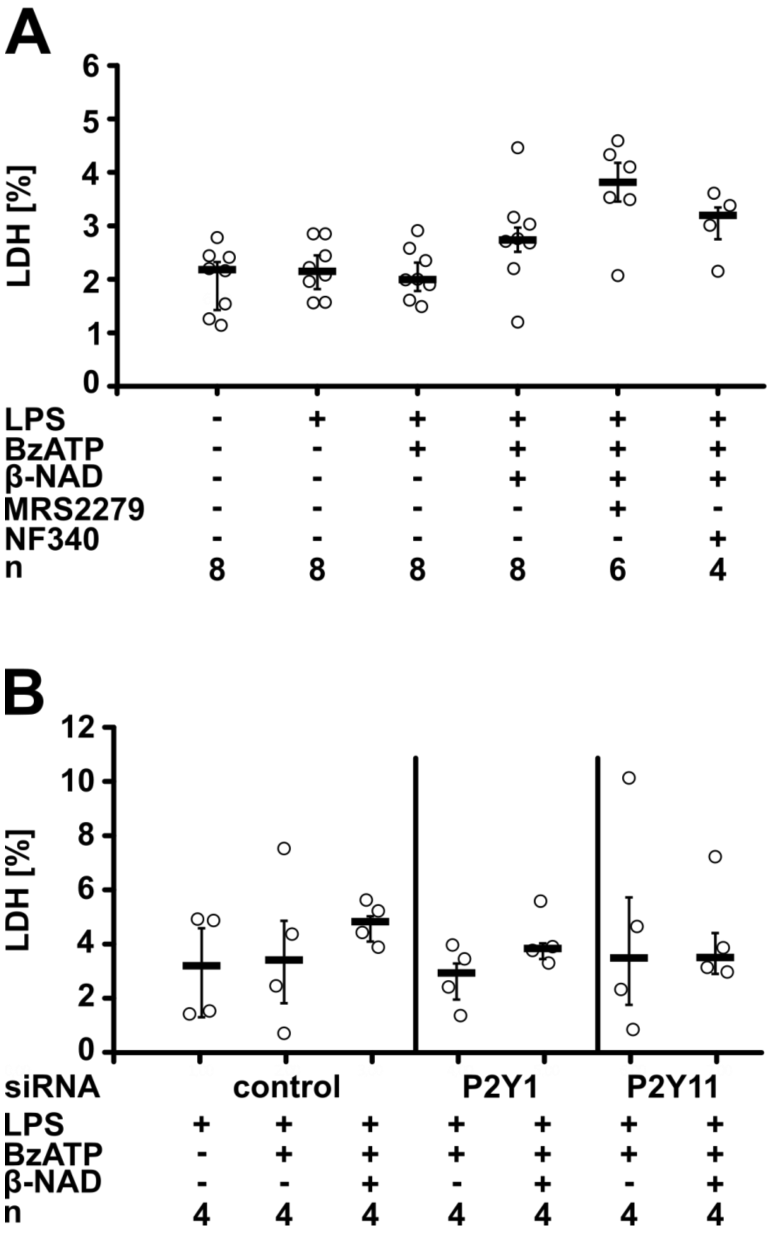

Figure S3. Estimation of cell death in experiments depicted in Figure 3. Human monocytic U937 cells were primed with LPS (1 µg/ml, 5 h) and stimulated with 2'(3')-O-(4-benzoylbenzoyl)adenosine-5'-triphosphate (BzATP; 100 µM, 30 min). β-NAD was applied together with BzATP at a concentration of 1 mM. The concentration of the cytoplasmic enzyme lactate dehydrogenase (LDH) was measured in the cell culture supernatants at the end of the experiments. LDH concentrations are indicated in % of the total cellular content of LDH in LPS-primed control cells. A) Experiments were performed in the presence or absence of the P2Y1 receptor antagonist MRS2279 (500 nM) or of the P2Y11 receptor antagonist NF340 (5 µM). B) U937 cells were transfected with control siRNA or with siRNA targeting P2Y1 or P2Y11 (P2RY1, P2RY11). No significant differences were induced by β-NAD ($p > 0.05$). Data are presented as individual data points, bars indicate median, whiskers encompass the 25th to 75th percentile, Kruskal-Wallis test followed by the Mann-Whitney rank sum test.

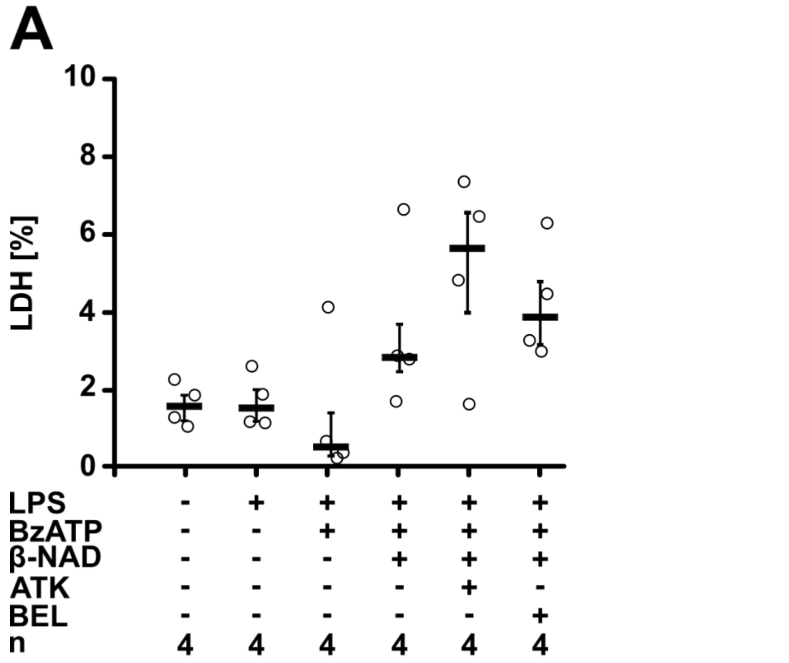

Figure S4. Estimation of cell death in experiments depicted in Figure 4. Human monocytic U937 cells were primed with LPS (1 µg/ml, 5 h) and activated with 2’(3’)-O-(4-benzoylbenzoyl)adenosine-5’-triphosphate (BzATP; 100 µM, 30 min). β-NAD was used at a concentration of 1 mM. The concentration of the cytoplasmic enzyme lactate dehydrogenase (LDH) was measured in the cell culture supernatants at the end of the experiments. LDH concentrations are indicated in % of the total cellular content of LDH in LPS-primed control cells. A) Experiments were performed in the presence or absence of the general inhibitor of PLA2 arachidonyl trifluoromethyl ketone (AKT; 50 µM) or of the more specific iPLA2 inhibitor bromoenol lactone (BEL; 50 µM). B) U937 cells were transfected with control siRNA or with siRNA targeting iPLA2β (PLA2G6). No significant differences were induced by β-NAD ($p > 0.05$). Data are presented as individual data points, bars indicate median, whiskers encompass the 25th to 75th percentile, Kruskal-Wallis test followed by the Mann-Whitney rank sum test.

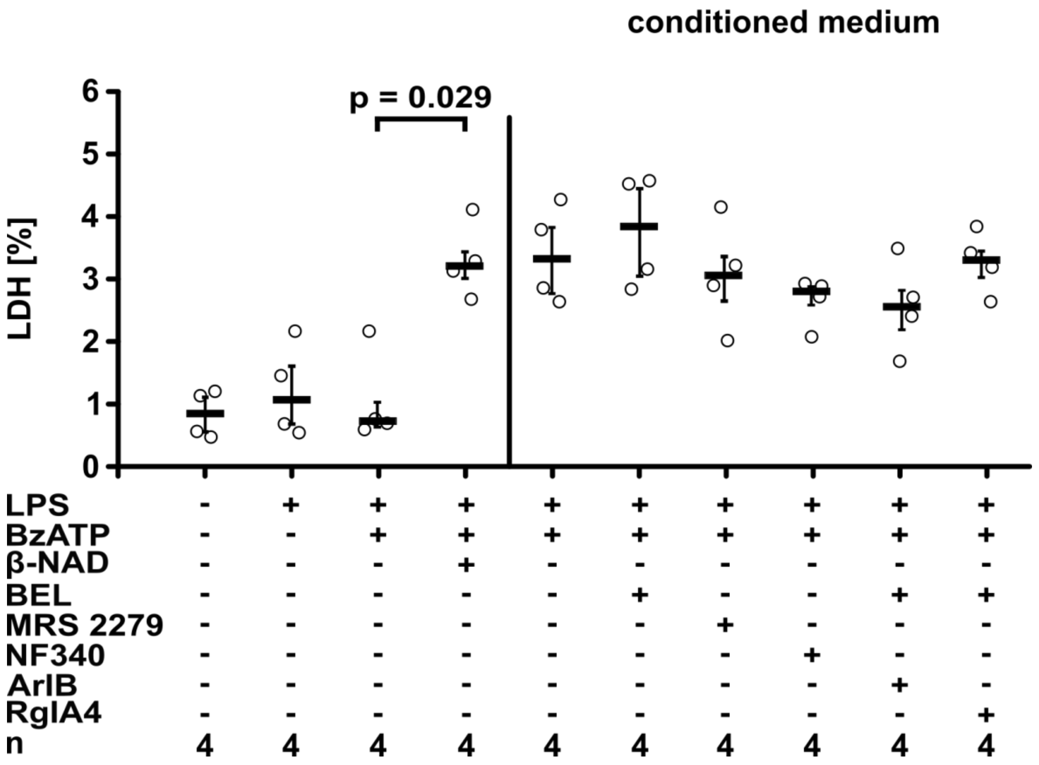

Figure S5. Estimation of cell death in experiments depicted in Figure 5. Human monocytic U937 cells were primed with LPS (1 µg/ml, 5 h) and treated with β-NAD (1 mM) for 30 min to produce conditioned medium. Thereafter, the conditioned medium was harvested and applied to another set of LPS-primed U937 cells together with 2’(3’)-O-(4-benzoylbenzoyl)adenosine-5’-triphosphate (BzATP; 100 µM) and incubated for 30 min. The P2Y1 antagonist MRS2279 (500 nM), the P2Y11 antagonist NF340 (5 µM), the iPLA2 inhibitor bromoenol lactone (BEL; 50 µM), the α7 nAChR antagonist α-conotoxin ArIB [V11L, V16D] (500 nM), or the α9α10 nAChR antagonist α-conotoxin RgIA4 (200 nM) were applied together with the conditioned medium. β-NAD (1 mM) was included in this experiment as a positive control in the absence of conditioned medium. The concentration of the cytoplasmic enzyme lactate dehydrogenase (LDH) was measured in the cell culture supernatants at the end of the experiments. LDH concentrations are indicated in % of the total cellular content of LDH in LPS-primed control cells. β-NAD induced a minor but statistically significant increase in LDH. A similar minor increase is seen, when conditioned medium was used. Data are presented as individual data points, bars indicate median, whiskers encompass the 25th to 75th percentile, Kruskal-Wallis test followed by the Mann-Whitney rank sum test.

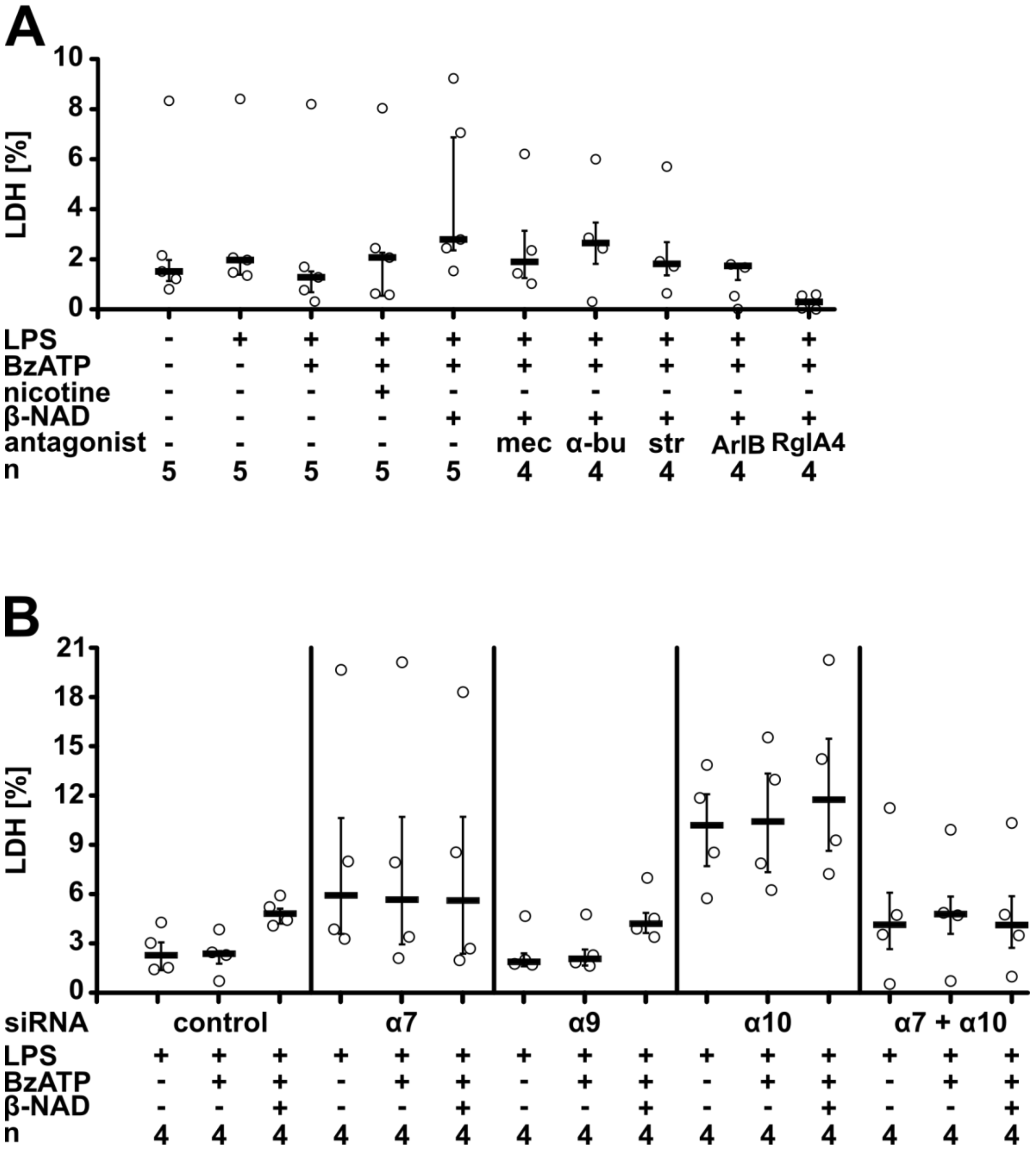

Figure S6. Estimation of cell death in experiments depicted in Figure 6. Human monocytic U937 cells were primed with LPS (1 µg/ml, 5 h) and activated with 2’(3’)-O-(4-benzoylbenzoyl)adenosine-5’-triphosphate (BzATP; 100 µM, 30 min). β-NAD was applied together with BzATP at a concentration of 1 mM. β-NAD (1 mM) was included in this experiment as a positive control in the absence of conditioned medium. The concentration of the cytoplasmic enzyme lactate dehydrogenase (LDH) was measured in the cell culture supernatants at the end of the experiments. LDH concentrations are indicated in % of the total cellular content of LDH in LPS-primed control cells. A) Nicotinic antagonists mecamylamine (mec; 100 µM), α-bungarotoxin (α-bu; 1 µM), strychnine (str; 10 µM), and ArIB [V11L, V16D] (500 nM) and RgIA4 (200 nM) were applied. B) U937 cells were transfected with control siRNA or with siRNA targeting nicotinic acetylcholine receptor subunits α7, α9, or α10 (*CHRNA7, CHRNA9, CHRNA10*). No significant differences were induced by β-NAD ($p > 0.05$). Data are presented as individual data points, bars indicate median, whiskers encompass the 25th to 75th percentile, Kruskal-Wallis test followed by the Mann-Whitney rank sum test.

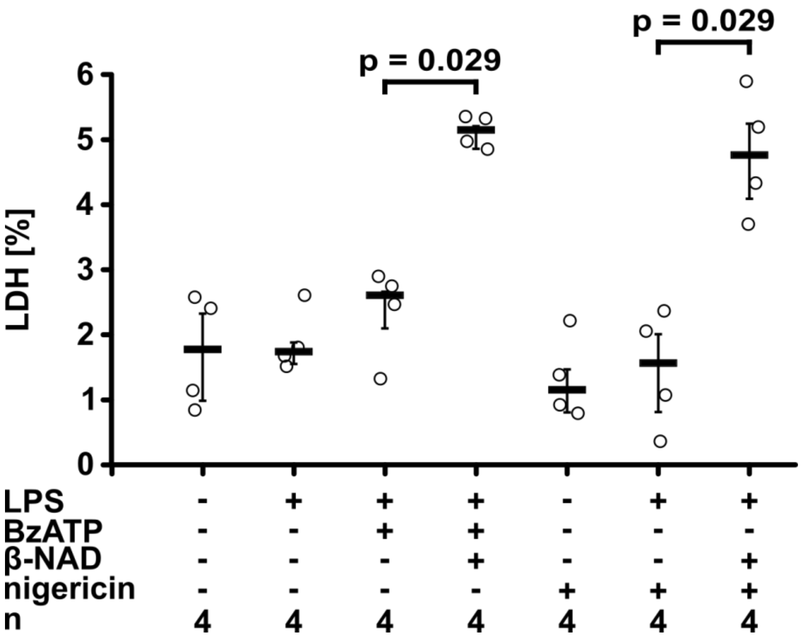

Figure S7. Estimation of cell death in experiments depicted in Figure 7A. Human monocytic U937 cells were primed with LPS (1 µg/ml, 5 h) and activated with 2'(3')-O-(4-benzoylbenzoyl)adenosine-5'-triphosphate (BzATP; 100 µM, 30 min) or nigericin (50 µM) in combination with apyrase (0.5 U/ml). β-NAD was used at a concentration of 1 mM. The concentration of the cytoplasmic enzyme lactate dehydrogenase (LDH) was measured in the cell culture supernatants at the end of the experiments. LDH concentrations are indicated in % of the total cellular content of LDH in LPS-primed control cells. β-NAD induced minor but statistically significant increases in LDH, whereas no significant differences were induced by nigericin ($p > 0.05$). Data are presented as individual data points, bars indicate median, whiskers encompass the 25th to 75th percentile, Kruskal-Wallis test followed by the Mann-Whitney rank sum test.

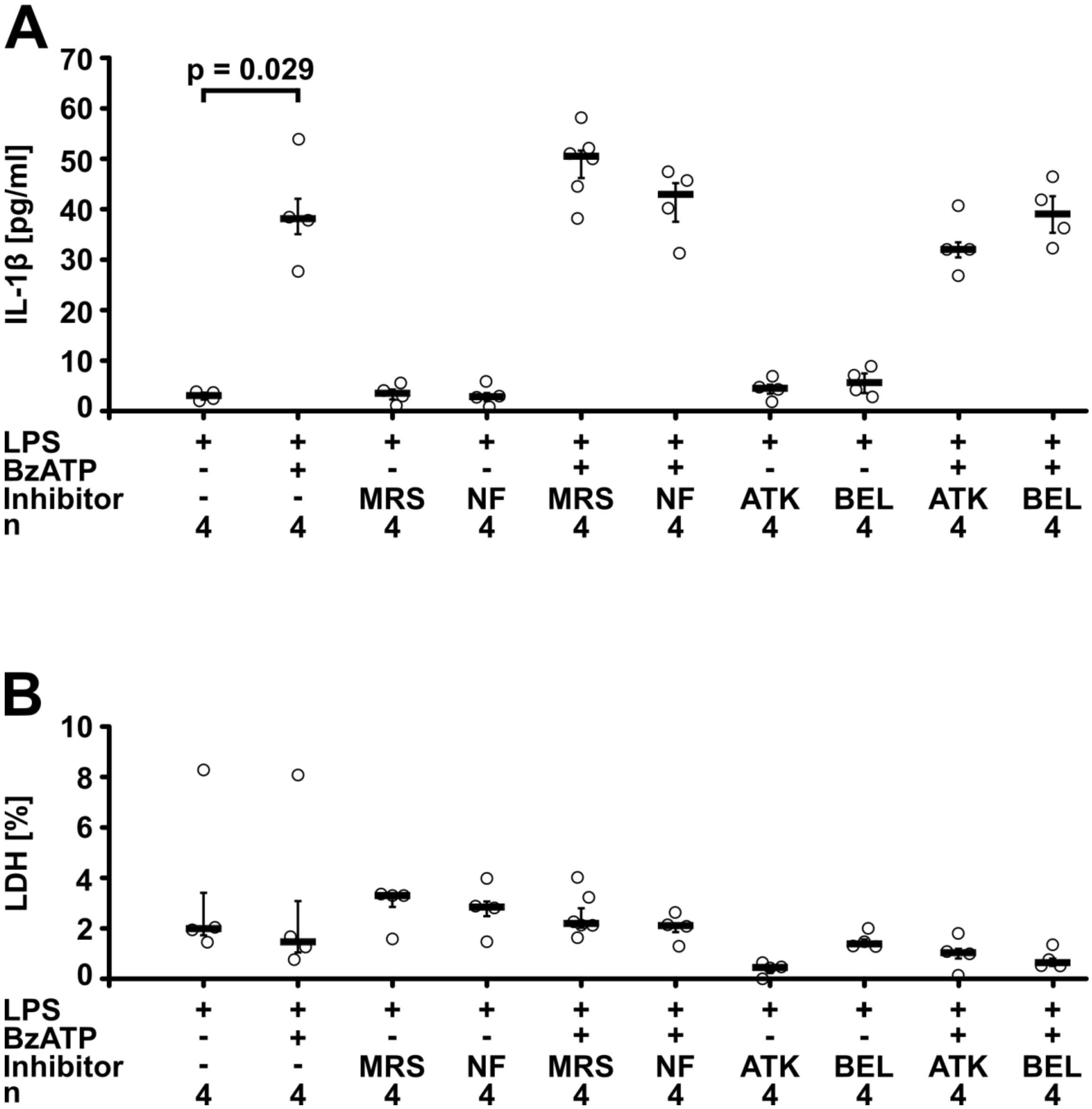

Figure S8: **P2Y receptor antagonists and phospholipase A2 inhibitors do not induce IL-1β release.** Human monocytic U937 cells were primed with LPS (1 µg/ml, 5 h) and stimulated with 2'(3')-O-(4-benzoylbenzoyl)adenosine-5'-triphosphate (BzATP; 100 µM, 30 min) as a positive control for the release of IL-1β. LPS-primed U937 cells were treated for 30 min with the P2Y1 receptor antagonist MRS2279 (MRS; 500 nM), the P2Y11 receptor antagonist NF340 (NF; 5 µM) or the inhibitors of phospholipase A2 arachidonyl trifluoromethyl ketone (AKT; 50 µM) or bromoenol lactone (BEL; 50 µM) in the absence or presence of BzATP. A) None of these compounds induced the release of IL-1β. The concentration of IL-1β in the cell culture supernatant was measured by ELISA. B) None of these compounds induced cell death as estimated by the concentration of the cytoplasmic enzyme lactate dehydrogenase (LDH) in the supernatants. LDH concentrations are indicated in % of the total cellular content of LDH in LPS-primed control cells. Data are presented as individual data points, bars indicate median, whiskers encompass the 25th to 75th percentile, Kruskal-Wallis test followed by the Mann-Whitney rank sum test.

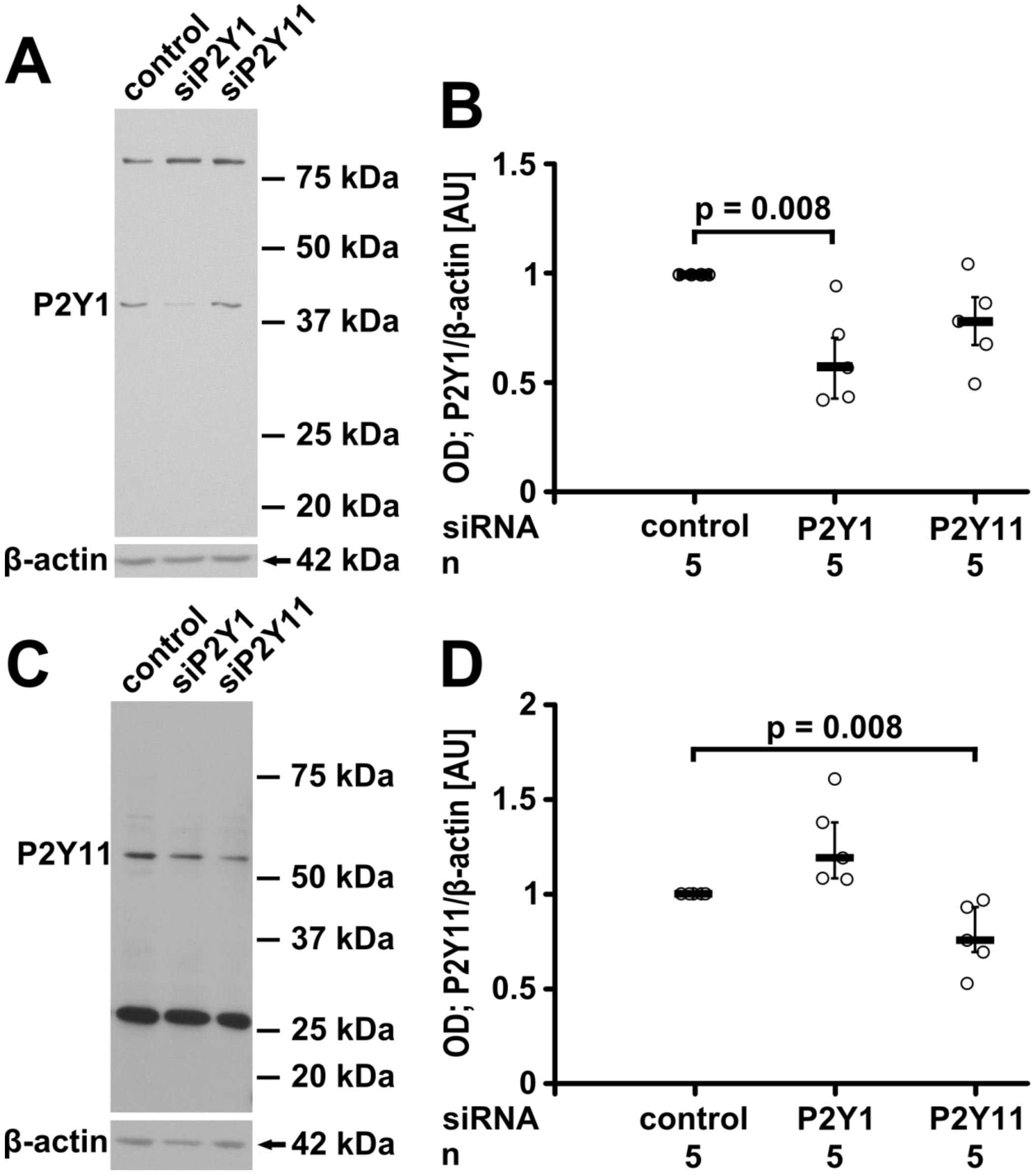

Figure S9: Transfection of siRNA efficiently and specifically reduces the expression of P2Y receptors. U937 cells were transfected with control siRNA or with siRNA targeting P2Y1 or P2Y11 (*P2RY1*, *P2RY11*). Two days after transfection, proteins were extracted, separated on 12% SDS polyacrylamide gels along with protein standards, blotted and detected with antibodies to P2Y1 (**A**) or P2Y11 (**C**). Detection of β-actin was included for normalization. (**B, D**) The optical density (OD) of the immuno-positive bands was measured and the ratio of the OD of the band corresponding to the receptor of interest and β-actin was formed. Values obtained for cells treated with siRNA targeting P2Y receptors were statistically compared to those transfected with control siRNA. The strong immuno-positive band with a molecular mass of about 27 kDa on the blot incubated with antibodies to P2Y11, is due to cross-reactivity with an irrelevant undetermined protein. Data are presented as individual data points, bars indicate median, whiskers encompass the 25th to 75th percentile, Kruskal-Wallis test followed by the Mann-Whitney rank sum test.

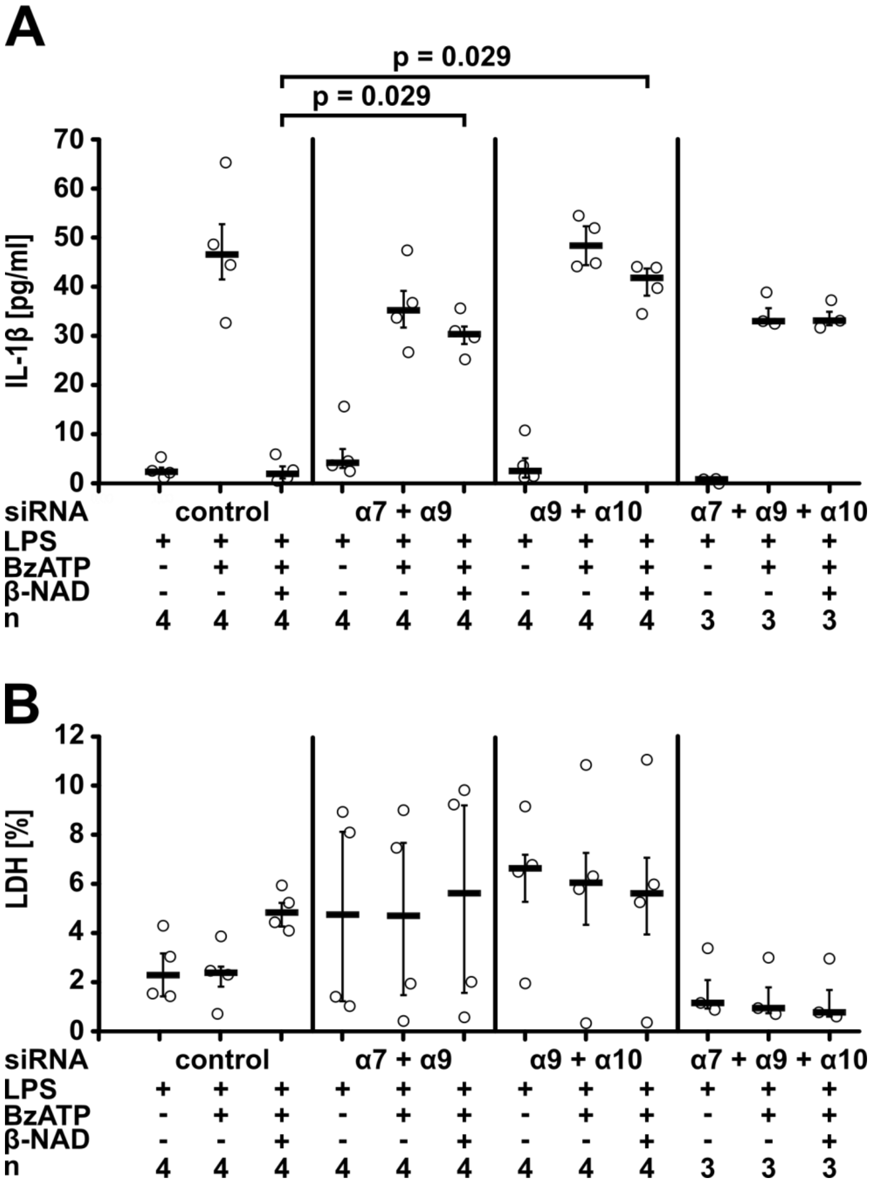

Figure S10: β-NAD signaling involves nicotinic acetylcholine receptors. Human monocytic U937 cells were transfected with control siRNA or with siRNA targeting nicotinic acetylcholine receptor subunits α7, α9, or α10 (*CHRNA7, CHRNA9, CHRNA10*). Nicotinic acetylcholine receptor subunits α7 and α9 or α9 and α10 were either targeted pairwise or a triple knock-down of three subunits was performed. Transfected U937 cells were primed with LPS (1 µg/ml, 5 h) and activated with 2’(3’)-O-(4-benzoylbenzoyl)adenosine-5’-triphosphate (BzATP; 100 µM, 30 min). β-NAD was used at a concentration of 1 mM. Results from cells transfected with control siRNA are included for comparison but were already shown in Fig. 5B. A) The concentration of IL-1β in the cell culture supernatant was measured by ELISA. B) Estimation of cell death as measured by the concentration of the cytoplasmic enzyme lactate dehydrogenase (LDH) in the supernatants. LDH concentrations are indicated in % of the total cellular content of LDH in LPS-primed control cells. Because of the low n-number in triple knock-down experiments, p-values were not determined. Data are presented as individual data points, bars indicate median, whiskers encompass the 25th to 75th percentile, Kruskal-Wallis test followed by the Mann-Whitney rank sum test.
